# Feedbacks, Bifurcations, and Cell Fate Decision-Making in the p53 System

**DOI:** 10.1371/journal.pcbi.1004787

**Published:** 2016-02-29

**Authors:** Beata Hat, Marek Kochańczyk, Marta N. Bogdał, Tomasz Lipniacki

**Affiliations:** 1 Institute of Fundamental Technological Research, Polish Academy of Sciences, Warsaw, Poland; 2 Department of Statistics, Rice University, Houston, Texas, United States of America; National Institutes of Health, UNITED STATES

## Abstract

The p53 transcription factor is a regulator of key cellular processes including DNA repair, cell cycle arrest, and apoptosis. In this theoretical study, we investigate how the complex circuitry of the p53 network allows for stochastic yet unambiguous cell fate decision-making. The proposed Markov chain model consists of the regulatory core and two subordinated bistable modules responsible for cell cycle arrest and apoptosis. The regulatory core is controlled by two negative feedback loops (regulated by Mdm2 and Wip1) responsible for oscillations, and two antagonistic positive feedback loops (regulated by phosphatases Wip1 and PTEN) responsible for bistability. By means of bifurcation analysis of the deterministic approximation we capture the recurrent solutions (i.e., steady states and limit cycles) that delineate temporal responses of the stochastic system. Direct switching from the limit-cycle oscillations to the “apoptotic” steady state is enabled by the existence of a subcritical Neimark—Sacker bifurcation in which the limit cycle loses its stability by merging with an unstable invariant torus. Our analysis provides an explanation why cancer cell lines known to have vastly diverse expression levels of Wip1 and PTEN exhibit a broad spectrum of responses to DNA damage: from a fast transition to a high level of p53 killer (a p53 phosphoform which promotes commitment to apoptosis) in cells characterized by high PTEN and low Wip1 levels to long-lasting p53 level oscillations in cells having PTEN promoter methylated (as in, e.g., MCF-7 cell line).

## Author Summary

Cancers are diseases of signaling networks. Transcription factor p53 is a pivotal node of a network that integrates a variety of stress signals and governs critical processes of DNA repair, cell cycle arrest, and apoptosis. Somewhat paradoxically, despite the fact that carcinogenesis is prevalently caused by p53 network malfunction, most of our knowledge about p53 signaling is based on cancer or immortalized cell lines. In this paper, we construct a mathematical model of intact p53 network to understand dynamics of non-cancerous cells and then dynamics of cancerous cells by introducing perturbations to the regulatory system. Cell fate decisions are enabled by the presence of interlinked feedback loops which give rise to a rich repertoire of behaviors. We explain and analyze by means of numerical simulations how the dynamical structure of the regulatory system allows for generating unambiguous single-cell fate decisions, also in the case when the cell population splits into an apoptotic and a surviving subpopulation. Perturbation analysis provides an explanation why cancer cell lines known to have vastly diverse expression levels of p53 regulators can exhibit a broad spectrum of responses to DNA damage.

## Introduction

The tumor suppressor p53 plays a pivotal role in cell growth control, DNA repair, cell cycle suppression and eventually in the initiation of apoptosis [1–4]. It serves as a node of a complex and extensive gene regulatory network that integrates a variety of stress signals. One of the most important ways of p53 activation is through DNA damage, which can be caused by, i.a., ionizing radiation (IR), UV radiation, hypoxia, heat shock, viral infection, or nutrient deprivation [1,5,6]. Exposure to IR inflicts DNA double strand breaks (DSBs), the most critical DNA lesions, which when unrepaired can lead to genomic instability resulting in either cell death or DNA mutations that can propagate to subsequent cell generations [7–9]. The p53 regulatory network provides mechanisms that suppress cell cycle until DNA is repaired or trigger apoptosis when DNA damage is too extensive to be repaired [4,7,10].

Unsurprisingly, mutations of the p53 gene (*TP53*) turn out to be the most frequent genetic changes in human cancers [11]. About half of all human cancer cells carry a mutation in *TP53*. In many other cancers, the genes which encode components of the p53 regulatory pathway are mutated [12,13]. The lack of functional p53 protein decreases the efficiency of DNA repair and allows mutated cells to evade apoptosis leading to the propagation of mutations and eventually to cancer development and progression [13,14]. Various malfunctions of the p53 regulatory pathway are determinants of tumor aggressiveness and p53 pathway components have become targets in anticancer therapies [14,15].

The primary molecular function of p53 is the regulation of transcription [16,17]. It controls expression of numerous genes which encode proteins of contradictory roles: pro-survival, cell cycle-suppressing, or pro-apoptotic [17]. Its ability to regulate expression of distinct sets of genes is controlled by posttranslational modifications [5]. In particular, p53 can assume two different phosphorylation states: p53_ARRESTER_ (when it is phosphorylated at Ser15 and Ser20 [5,18,19]) and p53_KILLER_ (when it is additionally phosphorylated at Ser46 [20,21]). In the arrester state, p53 triggers transcription of its main inhibitor, E3 ubiquitin ligase Mdm2 [22], cell cycle-suppressing protein p21 [23], and pro-survival phosphatase Wip1 [24,25]. In the killer state, p53 triggers transcription of pro-apoptotic phosphatase PTEN [26] and another pro-apoptotic protein, Bax [27].

In unstressed cells p53 remains inactive and is kept at a low level through constant proteasome-mediated degradation, regulated by Mdm2 [28–30]. p53 is activated by kinase ATM, which is rapidly phosphorylated in response to DNA damage [31,32]. The DNA damage detection system is sensitive; a handful of DSBs is sufficient for ATM and p53 activation; 1 Gy of irradiation induces about 35–40 DSBs [8,33]. ATM phosphorylates p53 at several residues, including Ser15 and Ser20, leading to its stabilization and activation as a transcription factor [19,34,35]. ATM phosphorylates also p53 inhibitor, Mdm2, promoting its degradation [36]. As a result, p53 concentration increases 3–10-fold within an hour after DNA damage [34]. Activity of p53 can be suppressed by growth factor stimulation leading to the activation of PI3 kinase (PI3K), which phosphorylates phosphatidylinositol bisphosphate (PIP2) into trisphosphate (PIP3) [37–40]. PIP3 allows for Akt recruitment to the plasma membrane where it can be activated via phosphorylation [41,42] by several kinases. Activated Akt phosphorylates Mdm2 at Ser166 and Ser186, allowing for its translocation to the nucleus where Mdm2 ubiquitinates p53 priming it for degradation [29,43].

The p53-regulated proteins comprise a complex regulatory network governed by multiple intertwined feedback loops spanning diverse time scales [44]. In addition to the primary p53 inhibitor, Mdm2, other key regulatory players in the network are two phosphatases: pro-survival Wip1 [24] and pro-apoptotic PTEN [26]. Wip1 attenuates signaling by dephosphorylating ATM [45] and inhibits apoptosis by facilitating dephosphorylation of p53_KILLER_ to the p53_ARRESTER_ form [46]. When DNA repair is accomplished, Wip1 enables the return to the pre-stress state [24,47]. PTEN mediates a positive feedback loop by inhibiting Akt and Mdm2, and by allowing p53_KILLER_ to rise to a high level and initiate apoptosis [48].

Dysregulation of the p53 pathway can occur as a consequence of gene amplification, gene loss, promoter methylation, or mutations which alter protein function. Gene amplification or inhibition are the major mechanisms which enhance expression of genes involved in cancer development and progression [49]. In particular, Wip1 amplification and overexpression have been found in multiple human cancers, predominantly in those that retain functional p53 [50–53] such as breast, lung, pancreas, bladder, and liver cancer, and in neuroblastomas [50,52,54,55]. Conversely, Wip1-knockout mice are partially resistant to oncogene-induced cancer development, indicating that the inhibition of Wip1 activity may be potentially beneficial for cancer therapy [45,52,56,57]. Decreased PTEN expression has been found in prostate, kidney, breast, bladder cancers, and in glioblastoma [58–60]. Cell lines with defective PTEN have alterations in the cell cycle regulation and a defective apoptotic response, which places PTEN among the tumor suppressors most commonly lost in human cancers. In contrast, overexpression of wild-type PTEN in cancer cells induces apoptosis and blocks cell-cycle progression [61,62]. Another dysregulated protein observed in many cancers is PI3K. The amplification of PI3K gene have been found in colorectal, gastric, hepatocellular, thyroid, breast, lung, and ovarian cancer, and in glioblastoma and acute leukemia [63–70].

The clinical data shows that differences between cancer cells can be partially explained by different expression levels of PTEN, Wip1, and PI3K. The majority of breast cancers reveals amplification of Wip1 and reduced level of PTEN, which appear to correlate with poor prognosis as well as increased resistance to γ radiation-based therapy and apoptosis [55,58].

A number of computational models have been constructed to investigate the regulatory mechanisms of the p53 pathway. Earlier models were focused on explaining the origin of oscillations of p53 and Mdm2 levels in response to IR [71–76], which were first observed in the cell population experiment on MCF-7 cells by Bar-Or et al. [71] and later in single-cell experiments by Lahav et al. [77] and Geva-Zatorsky et al. [73]. Recently, the effort has been shifted to connecting p53 dynamics with cell fate decisions [75,78–85]. For the sake of conciseness of the Introduction, an overview of p53 modeling results cited in this paragraph is provided in S1 Text.

In this study, we focus on the p53 regulatory core to demonstrate that the structure of the p53 regulatory network is such that for a broad range of parameters at persistent DNA damage the limit cycle oscillations of p53_ARRESTER_ (as well as p53_KILLER_) coexist with the steady state characterized by a high level of p53_KILLER_. The detailed bifurcation analysis shows that the direct switch between oscillations and high steady state is enabled by the existence of a Neimark—Sacker bifurcation. These two recurrent solutions delineate temporal responses of the system, which can be interpreted unambiguously by two slaved modules which control cell cycle arrest and apoptosis. This is confirmed by stochastic simulations of the model dynamics, showing clear separation of cells into surviving and apoptotic subpopulations about 30 hours after DNA damage.

## Results

### Overview of the model structure

**Fig 1:**
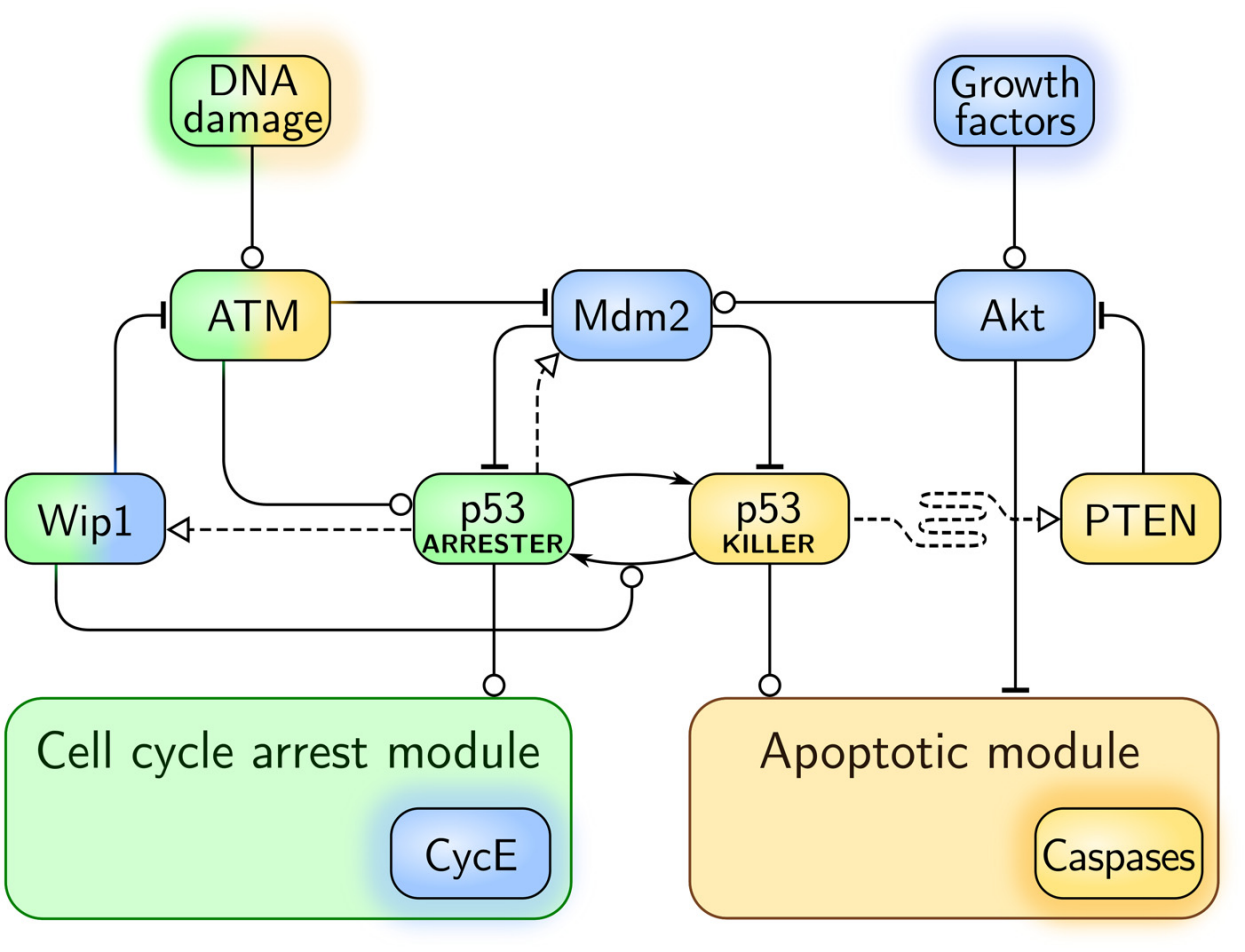
Simplified scheme of the model. Arrow-headed dashed lines indicate positive transcriptional regulation, arrow-headed solid lines—protein transformation, circle-headed solid lines—positive influence or activation, hammer-headed solid lines—inhibitory regulation. Pro-survival and cell cycle-promoting proteins are represented with blue boxes, pro-apoptotic proteins with yellow boxes, proteins involved in cell cycle arrest with green boxes. Details of the cell cycle arrest module and the apoptotic module are shown in Figs 2 and 3 respectively.

The proposed model consists of three modules: the core module, the cell cycle arrest module, and the apoptotic module. In Fig 1 we show a simplified scheme which summarizes the key feedback loops present in the core module. The detailed scheme with all model components is provided in S1 Fig. The regulatory pathway considered consists of 42 species (see Tables A and B in S1 Text), coupled by 74 reactions parametrized by 97 reaction rate coefficients (see Table C in S1 Text). The stochastic model is equivalent to a Markov process while its deterministic approximation is represented by ODEs. The levels of all substrates in stochastic as well as in deterministic simulations are expressed in the numbers of molecules per cell (mlcs/cell).

#### Core module

We consider the p53 system responses to DNA damage introduced by IR. DNA damage leads to the activation of ATM. Active ATM phosphorylates p53 inhibitor, Mdm2, priming it for faster degradation. It also stabilizes and activates p53 by phosphorylating it to one of its active phosphoforms, p53_ARRESTER_. This phosphoform can be further phosphorylated (at Ser46) to the p53_KILLER_ form. p53_ARRESTER_ induces synthesis of Mdm2 and Wip1, a phosphatase which dephosphorylates ATM as well as p53_KILLER_ to the p53_ARRESTER_ form.

p53_KILLER_ activates expression of another phosphatase, PTEN, which mediates the slow positive feedback loop which stabilizes the level of p53. PTEN indirectly suppresses Akt catalytic activity, which is required to phosphorylate Mdm2 in order to enable its nuclear import. As a result, accumulation of PTEN leads to the accumulation of Mdm2 in cytoplasm and therefore physically disconnects nuclear p53 from its inhibitor, leading to the stabilization of p53_KILLER_ and p53_ARRESTER_ at high levels. PTEN action is opposed by the growth and survival factors which lead to Akt activation.

Cell cycle arrest module is controlled by p53_ARRESTER_. This phosphoform activates transcription of p21 which suppresses cell cycle by inhibiting cyclin E (CycE). The apoptotic module is controlled by p53_KILLER_, which activates transcription of pro-apoptotic protein Bax, and by Akt, which suppresses the apoptosis by phosphorylation of 14-3-3 protein.

Among more than ten feedbacks present in the system the following four are of key functional importance:

- **F1** Negative feedback p53_ARRESTER_ → Mdm2 –| p53: p53_ARRESTER_ activates transcription of Mdm2 which ubiquitinates p53 inducing its rapid degradation. This feedback maintains homoeostasis in unstimulated cells and enables oscillations of p53 level upon DNA damage.
- **F2** Negative feedback ATM → p53_ARRESTER_ → Wip1 –| ATM: ATM activates p53 to the p53_ARRESTER_ form which activates transcription of Wip1, which in turn deactivates ATM. This feedback leads to recurrent ATM inhibition and initiation upon DNA damage enabling persistent oscillations of the p53 level.
- **F3** Positive feedback p53_ARRESTER_ → Wip1 → p53_ARRESTER_: p53_ARRESTER_ activates transcription of Wip1, which dephosphorylates p53_KILLER_ to p53_ARRESTER_. This feedback stabilizes p53 in the arrester state and attenuates accumulation of p53_KILLER_ preventing/postponing apoptosis.
- **F4** Positive feedback p53_KILLER_ → PTEN –| Akt → Mdm2 –| p53_KILLER_: p53_KILLER_ activates transcription of PTEN which (indirectly) deactivates Akt; Akt phosphorylates Mdm2 allowing for its import to the nucleus where Mdm2 ubiquitinates p53 inducing its rapid degradation. This feedback stabilizes the state of a high p53_KILLER_ and low Akt activity, and thus induces apoptosis.

Two negative feedback loops, F1 and F2, associated with time delays introduced by the mRNA transcription, protein translation, and nuclear import, induce oscillatory responses upon DNA damage. As shown experimentally in [82] and demonstrated in analysis in S2 Fig the system without the negative feedback to ATM does not exhibit oscillations. The two positive feedbacks, F3 and F4, introduce bistability. These two feedbacks, mediated by Wip1 and PTEN phosphatases, act antagonistically. The Wip1-mediated loop (F3) prevents p53_KILLER_ accumulation (by dephosphorylating p53_KILLER_ to p53_ARRESTER_) while the PTEN-mediated loop (F4) “supports” p53_KILLER_ accumulation (by indirect inhibition of Mdm2). The PTEN loop results from double negation and involves several intermediates (PTEN, PIP2/PIP3, Akt, Mdm2), therefore its dynamics is slower. As a result, even in cells with extensive DNA damage, p53_KILLER_ accumulation (and thus apoptosis) is postponed, granting the cell time which can be used for DNA repair. When DNA repair is accomplished before signal passes through the PTEN loop, the cell survives. However, when the DNA damage is severe so that its repair takes longer time, the PTEN-mediated feedback takes over and the cell commits to apoptosis.

#### Cell cycle arrest module

**Fig 2:**
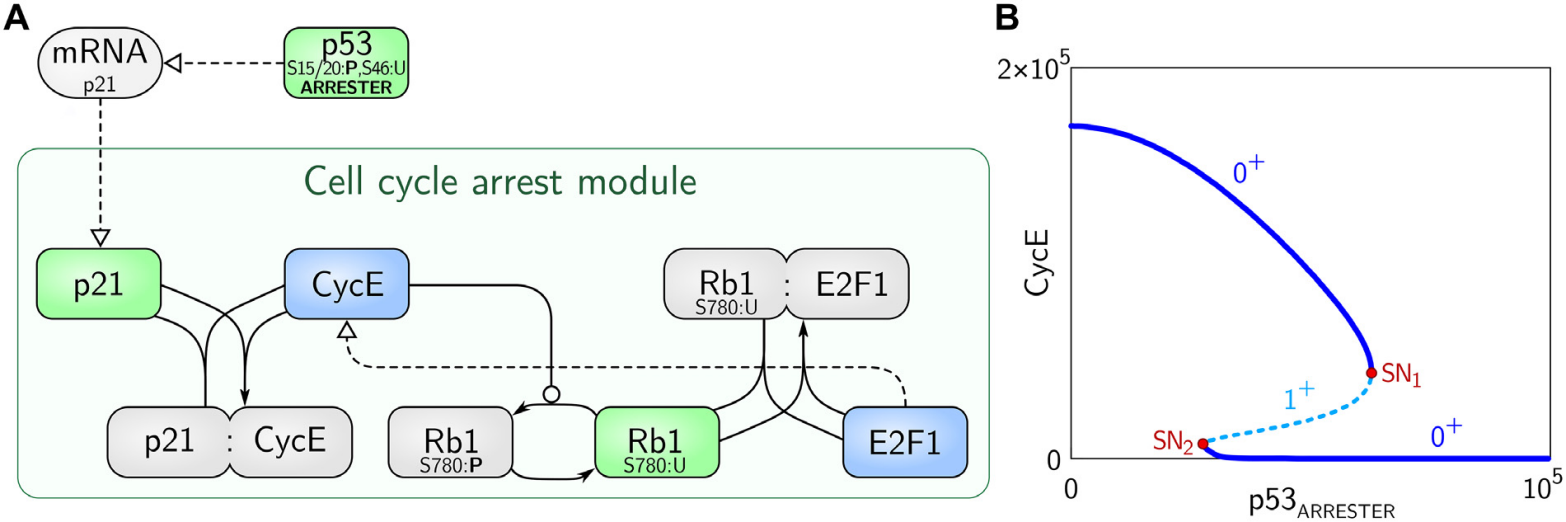
Scheme and the bifurcation diagram for the cell cycle arrest module. (A) Scheme: cell cycle-arresting proteins are represented with green boxes, proteins promoting cell cycle with blue boxes, other proteins or complexes with gray boxes. Bold ‘P’ denotes a phosphorylated protein residue. The arrow notation is same as in Fig 1. (B) Bifurcation diagram of CycE vs. p53_ARRESTER_ (bifurcation parameter). The stable and unstable steady states are indicated by solid and dashed lines, respectively. SN_1_ and SN_2_ denote saddle-node bifurcations. The number of eigenvalues with positive real parts is either one (1^+^) for the unstable steady state or zero (0^+^) for stable steady states.

p53_ARRESTER_ induces expression of p21, a protein which suppresses cell cycle progression to allow for DNA repair (Fig 2A). p21 directly binds and suppresses CycE which plays a critical role in the transition from G1 to S phase, and is considered a marker of cell cycle progression in the model [23]. CycE, synthesis of which is positively regulated by E2F1 (and several other transcription factors from the E2F family), can inactivate Rb1 protein by inducing its phosphorylation at Ser780. Phosphorylated Rb1 can no longer inhibit E2F1. This creates a positive feedback loop (based on double negation) in which CycE suppresses the inhibitor of its own transcription factor. This mode of regulation introduces bistability and allows for switch-like exit from and return to the cell cycle progression. Cell cycle progresses at a low level of p21 (i.e., when most of CycE is free). As p21 level increases the fraction of free CycE decreases, leading to dephosphorylation of Rb1 and inhibition of E2F1. At some level of p21 the system rapidly transits to the state in which most of E2F1 is inhibited and the level of CycE is close to zero (Fig 2B).

The analysis of the cell cycle arrest module shows that this subsystem exhibits bistability for a range of p53_ARRESTER_ levels (Fig 2B). The low level of p53_ARRESTER_ corresponds to a high level of CycE at which the cell cycle can progress. As the level of p53_ARRESTER_ increases, the level of CycE decreases until the stable state loses its stability in the bifurcation point SN_1_ and the system switches to the lower stable steady state in which the cell cycle is suppressed. When p53_ARRESTER_ decreases from a high level, the system undergoes SN_2_ bifurcation and the cell cycle may progress again.

#### Apoptotic module

**Fig 3:**
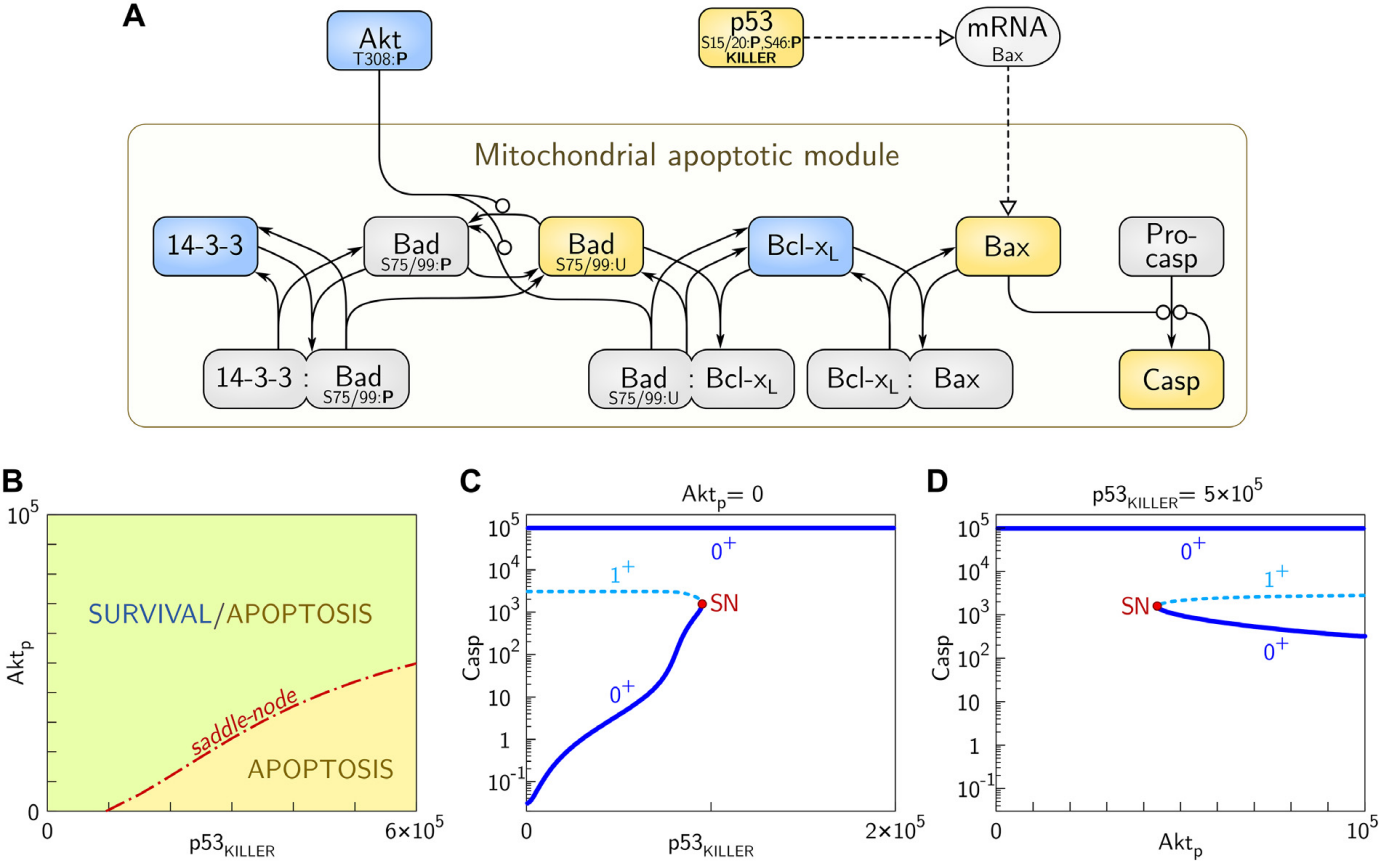
Scheme and the bifurcation diagrams for the apoptotic module. (A) Scheme: pro-survival proteins are in blue boxes, pro-apoptotic proteins are in yellow boxes, other proteins or protein complexes are in gray boxes. The arrow notation is same as in Fig 1. (B) Saddle—node bifurcations line in the (p53_KILLER_, Akt_p_)-plane (2-D bifurcation diagram) separates the region of parameters for which apoptotic and survival states coexist from the region for which only the apoptotic state exists. (C) Bifurcation diagram for Casp vs. p53_KILLER_ (bifurcation parameter) for Akt_p_; the saddle-node bifurcation point SN is (p53_KILLER_, Casp_bif_) ≅ (0.95×10^5^, 1.5×10^3^). (D) Bifurcation diagram for Casp vs. Akt_p_ (bifurcation parameter) for p53_KILLER_ ≅ 5×10^5^; the saddle-node bifurcation point SN is (Akt_p_, Casp_bif_) ≅ (4.4×10^4^, 1.5×10^3^). The solid and dashed lines correspond respectively to the stable and unstable steady states. The number of eigenvalues with positive real parts is either one (1^+^) for the unstable steady state or zero (0^+^) for stable steady states. Notice the logarithmic scale on vertical axes in (C) and (D).

The detailed description and analysis of the apoptotic module is contained in our recent paper by Bogdał et al. [86]. In this module (Fig 3A) we consider two inputs: pro-survival input, strength of which increases with the level of phosphorylated Akt (Akt_p_), and pro-apoptotic, strength of which increases with the level of p53_KILLER_. Non-apoptotic cells are characterized by a relatively high level of Akt_p_ and the lack or a very low level of p53_KILLER_. The following two signals promote apoptosis: (1) suppression of pro-survival Akt, i.e., decrease of the Akt_p_ level, and (2) increase of the p53_KILLER_ level.

In non-apoptotic cells the apoptosis is suppressed since the apoptotic effector, Bax, is neutralized through sequestration by pro-survival Bcl-x_L_. This is possible because the other pro-apoptotic protein, Bad, that can bind Bcl-x_L_, remains phosphorylated by Akt_p_ and sequestered by pro-survival protein 14-3-3. Dephosphorylation of Akt (which is considered here as pro-apoptotic signal) leads to the dephosphorylation of Bad and its release from the complex with 14-3-3. Subsequently, Bad binds to Bcl-x_L_ displacing Bax, which accumulates in the mitochondrial membrane, leading eventually to the release of cytochrome c, which initiates caspase activation (active both initiator and executioner caspases are collectively denoted as Casp). In the model, the step of cytochrome c release is omitted; instead, we assume that Bax activates Casp directly. The other pro-apoptotic signal comes from p53_KILLER_, accumulation of which triggers Bax transcription and Bax protein accumulation.

In Fig 3B we analyze how cell fate decisions depend on the levels of p53_KILLER_ and Akt_p_. In the (p53_KILLER_, Akt_p_)-plane the saddle—node bifurcations line separates the region where survival coexists with apoptosis from the apoptotic region. The transition from survival to apoptosis requires crossing the saddle—node line, that is possible only when the level of p53_KILLER_ increases and, simultaneously, a fraction of phosphorylated Akt (Akt_p_) drops. Therefore, the apoptotic module integrates information about Akt_p_ and p53_KILLER_ levels in the way similar to the AND logic gate (meaning that simultaneous dephosphorylation of Akt and build-up of p53_KILLER_ are needed to trigger apoptosis). In our previous study [86], we demonstrated that the system can behave digitally, either as the logic gate AND, or as the logic gate OR, depending on the levels of Bad and Bcl-x_L_. Gate AND arises for high levels of Bcl-x_L_ and low levels of Bad. The OR gate (when apoptosis may be initiated by one of the two apoptotic signals) arises for high Bad and low Bcl-x_L_ levels.

In Fig 3C and 3D we show bifurcation diagrams of the apoptotic switch with p53_KILLER_ (Fig 3C) and Akt_p_ (Fig 3D) considered bifurcation parameters. The model predicts that for Akt_p_ = 0 and p53_KILLER_ < 10^5^ the cell can exist either in the apoptotic or in surviving steady state, characterized by a high and a low Casp levels, respectively (Fig 3C). At p53_KILLER_ ≈ 10^5^ the system undergoes the saddle—node bifurcation (in which Casp ≈ 1.5 × 10^3^) in which the surviving state vanishes. In Fig 3D we show that for a high level of p53_KILLER_ (i.e., p53_KILLER_ ≈ 5 × 10^5^) the system exhibits bistability for Akt_p_ > 4.5 × 10^4^. At Akt_p_ ≈ 4.5 × 10^4^ the system undergoes the saddle—node bifurcation, and for Akt_p_ < 4.5 × 10^4^ only the apoptotic state exists. The bifurcation diagrams shown in Fig 3C and 3D imply that the caspase activation switch (in contrast to the cell cycle arrest switch) is irreversible, i.e., there is no possibility to return from the apoptotic to the surviving state (even after p53_KILLER_ drops to zero or Akt_p_ grows to its maximal value). The structure of the bifurcation diagram of Casp vs. p53_KILLER_ resembles qualitatively the bifurcation diagram of Casp vs. Bax analyzed in our earlier work [86].

### Bifurcation analysis of the p53 core module

In this section we focus on the discrimination of possible responses to DNA damage. In the bifurcation analysis we assume that the number of DSBs remains constant and equal to 100, which is considered as severe DNA damage. Bifurcation analysis allows us to determine the recurrent solutions (i.e., steady states and limit cycles) that delineate time-dependent responses to DNA damage. Since the two positive feedback loops are controlled by phosphatases Wip1 and PTEN, expression levels of which are varied substantially among cancer cells, we choose Wip1 and PTEN synthesis rates as bifurcation parameters. Additionally, PTEN has slow dynamics with respect to the other components and its gradual accumulation after DNA damage controls the behavior of the whole system. As a result, the system behavior can be predicted by bifurcation analysis in which PTEN is a bifurcation parameter.

**Fig 4:**
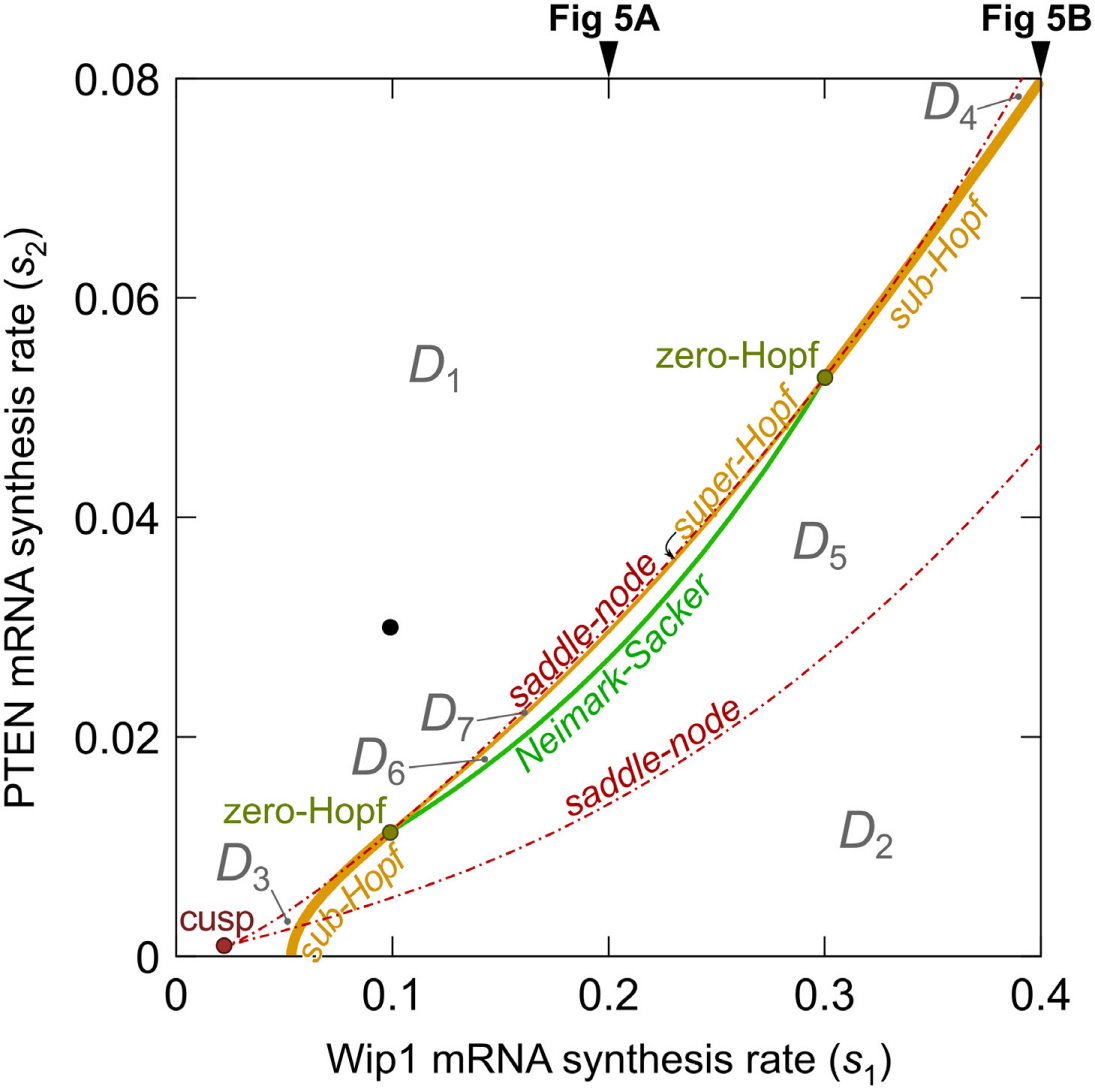
Two-dimensional bifurcation diagram showing various types of bifurcation lines and points in the (Wip1 synthesis rate, PTEN synthesis rate)-plane. The black dot indicates nominal values of parameters *s*_1_ and *s*_2_. The bifurcation lines divide the parameter domain into seven subdomains *D*_1_,…,*D*_7_. The recurrent solutions in each of these domains are given in main text. Bifurcation diagrams in Fig 5A and 5B show the recurrent solutions obtained for *s*_1_ = 0.2 and *s*_1_ = 0.4, as indicated by black arrows, and varied *s*_2_.

In Fig 4 we show the two-dimensional bifurcation diagram in the (s_1_, s_2_)-plane, where s_1_ and s_2_ are Wip1 and PTEN synthesis rates, respectively. As shown, the system involves three types of bifurcations: Hopf, saddle-node, and Neimark—Sacker. In the 2-D diagram, Neimark—Sacker bifurcations line arises at one zero—Hopf point (s_1_, s_2_) ≈ (0.1, 0.012) and vanishes at another zero—Hopf point (s_1_, s_2_) ≈ (0.3, 0.053). Between these points the Hopf bifurcations are supercritical, while outside them they are subcritical. The supercritical Hopfs line lies between the saddle—node line and the Neimark—Sacker bifurcations line. The saddle—node lines arise in a cusp point (s_1_, s_2_) ≈ (0.02, 0.001). The bifurcation lines divide the (s_1_, s_2_)-plane into subregions in which the system exhibits different behaviors. In short, the system is oscillatory in the parameter subdomain to the right of the Hopf bifurcations line, i.e., oscillations can arise due either increase of Wip1 synthesis or decrease of PTEN synthesis. The bistability (understood here as either the coexistence of two stable steady states or coexistence of one stable steady state and one stable limit cycle) can arise between two saddle—node lines.

The bifurcation lines divide the (s_1_, s_2_)-plane into seven subdomains characterized by different recurrent solutions:

*D*_1_: monostability (one stable steady state),

*D*_2_: oscillatory (stable limit cycle and one unstable steady state),

*D*_3_: typical bistability (two stable steady states and one unstable steady state),

*D*_4_: typical bistability (two stable steady states and one unstable steady state),

*D*_5_: atypical bistability (the stable limit cycle coexists with one stable steady state and two unstable steady states),

*D*_6_: monostability (one stable steady state, two unstable steady states and one unstable limit cycle),

*D*_7_ (the tiny region between supercritical Hopf line and upper saddle—node line): monostability (one stable steady state and two unstable steady states).

We chose (s_1_, s_2_) = (0.1, 0.03) as nominal model values for the Wip1 and PTEN mRNA synthesis rates. According to Fig 4, (s_1_, s_2_) = (0.1, 0.03) lies in domain *D*_1_, in which the system with persistent DNA damage has only one solution, the stable steady state. As we will see, this state is characterized by the level of p53_KILLER_ high enough to trigger apoptosis.

**Fig 5:**
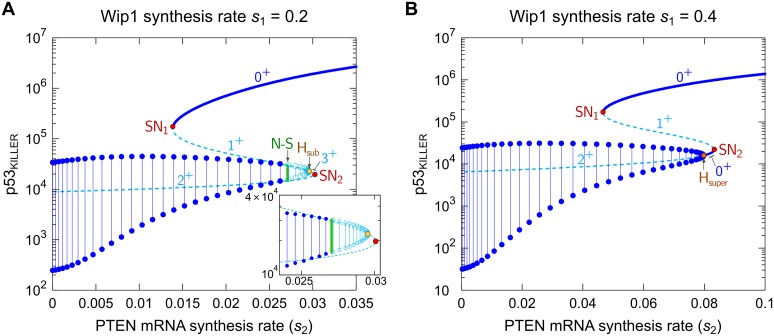
Bifurcation diagram of p53_KILLER_ vs. PTEN mRNA synthesis rate (*s*_2_). (A) Wip1 synthesis rate *s*_1_ = 0.2, (B) *s*_1_ = 0.4. The stable and unstable steady states are indicated by solid and dashed lines, respectively. Ranges of stable and unstable limit cycles are indicated by dark and light blue lines, respectively; dots and open circles are the maxima and minima of the stable and unstable limit cycles, respectively. Green vertical line in (A) shows Neimark—Sacker bifurcation (N—S). Red dots mark saddle-node bifurcations (SN_1_, SN_2_), orange dots mark supercritical Hopf (H_super_) and subcritical Hopf (H_sub_) bifurcations. The numbers in format *n*^+^ adjacent to the steady state lines denote the number of eigenvalues with the positive real parts. Notice the logarithmic scale on vertical axes.

In Fig 5 we show bifurcation diagrams of p53_KILLER_ as a function of the PTEN synthesis rate, s_2_. We consider two values of s_1_, s_1_ = 0.2 and s_1_ = 0.4. The character of the bifurcation diagram for s_1_ = 0.2 is qualitatively similar to that for nominal s_1_ = 0.1, however it is visually clearer since the distance between Neimark—Sacker, Hopf, and saddle—node bifurcations is larger. For s_1_ = 0.4 (Fig 5B) the bifurcation diagram is qualitatively different from that for s_1_ = 0.1 and s_1_ = 0.2 (Fig 5A).

As shown in Fig 4, with s_2_ increasing (for fixed s_1_ = 0.2) the system proceeds sequentially through five subdomains in the (s_1_, s_2_)-plane: *D*_2_, *D*_5_, D_6_, *D*_7_, and *D*_1_. Fig 5A visualizes details of recurrent solutions along this line. In the description below we focus on stable steady states and limit cycles because these solutions shape time-dependent responses. For s_2_ < s_SN1_, i.e., in subdomain *D*_2_, the system has a single stable limit cycle. This solution is characterized by oscillations of the levels of p53_KILLER_ and p53_ARRESTER_. Amplitude of p53_ARRESTER_ oscillations is high enough to suppress the cell cycle, but the level of p53_KILLER_ remains below the apoptotic threshold.

At s_2_ = s_SN1_ the first saddle—node bifurcation arises, and for s_2_ ∈ (s_SN1_; s_N−S_), i.e., in *D*_5_ subdomain, the system exhibits atypical bistability in which the stable limit cycle coexists with the stable steady state. This steady state is characterized by the p53_KILLER_ level high enough to trigger apoptosis. Therefore, in this parameter range the apoptotic state and cell cycle arrest state coexist. At s_2_ = s_N−S_ the subcritical Neimark—Sacker bifurcation arises, in which the stable limit cycle loses its stability merging with an unstable invariant torus, and for s_2_ > s_N−S_ the only stable recurrent solution is the stable steady state associated with apoptosis. Therefore, as parameter s_2_ increases (which is associated with the increase of the PTEN level), the cell with damaged DNA at s_2_ = s_N−S_ switches abruptly to apoptosis.

This biologically plausible behavior, allowing for unambiguous cell fate decisions, is associated with the specific bifurcation structure involving the subcritical Neimark—Sacker bifurcation. Then the cycle vanishes at ${\text{s}}_{2}={\text{s}}_{{\text{H}}_{\text{super}}}$ in the supercritical Hopf bifurcation (see the Fig 5A insert). Subsequently, two remaining unstable steady states (one with 3 eigenvalues of positive real parts), and the other (with 2 eigenvalues of positive real parts) “annihilate” in s_2_ = s_SN2_. Let us notice that unstable steady states in the analyzed diagram have at most 3 eigenvalues with positive real parts and the unstable cycle has 2 Floquet multipliers of moduli larger than 1. This implies that despite the system has complex structure which involves numerous feedback loops, its dynamics is essentially 3-dimensional, meaning that locally there exists a 3-dimensional attracting manifold.

A qualitatively different is the bifurcation diagram depicting the recurrent solutions along line s_1_ = 0.4 with increasing s_2_ (Fig 5B), i.e., when the system proceeds sequentially through *D*_2_, *D*_5_, *D*_4_, and *D*_1_ subdomains (shown in Fig 4). Similarly to the previous case for s_2_ < s_SN1_, the system has the stable limit cycle (cell cycle arrest state). Then, at s_2_ = s_SN1_, the first saddle—node bifurcation arises, and for ${\text{s}}_{2}\;\epsilon \;\left({\text{s}}_{\text{SN}1};{\text{s}}_{{\text{H}}_{\text{sub}}}\right)$ the stable limit cycle coexists with the stable steady state (apoptotic state). However, in contrast to bifurcation diagram shown in Fig 5A, the stable limit cycle does not lose its stability, but it is replaced by a steady state at ${\text{s}}_{2}={\text{s}}_{{\text{H}}_{\text{sub}}}$ through the subcritical Hopf bifurcation. In this steady state, p53_KILLER_ does not exceed the apoptotic threshold. This steady state annihilates with an unstable steady state at s_2_ = s_SN2_ in the second saddle—node bifurcation. As in the previous case, for s_2_ > s_SN2_ the system has a single recurrent solution, the stable steady state associated with apoptosis.

We think that the bifurcation structure in Fig 5A reflects the experimental observations better than that of Fig 5B, suggesting that upon DNA damage the level of p53 either oscillates at a relatively low amplitude, or builds up to the high level in which apoptosis is triggered. Therefore, for further analysis we assume s_1_ = 0.1, resulting in a bifurcation diagram as in Fig 5A. The bifurcation diagrams corresponding to s_1_ = 0.1 (or qualitatively equivalent s_1_ = 0.2) calculated with respect to Wip1, PI3K, and the number of DSBs are shown in S3 Fig. Qualitatively, diagrams with respect to Wip1 and PI3K are mirror images of the bifurcation diagram with respect to PTEN (Fig 5A), confirming the antagonistic action of Wip1 and PTEN. The antagonism of PI3K and PTEN is straightforward because PI3K phosphorylates PIP2 to PIP3, while PTEN dephosphorylated PIP3 to PIP2. The bifurcation diagram with respect to DNA damage (assumed, for this diagram, to be persistent) is similar to the bifurcation diagram with respect to PTEN, but the limit cycle oscillations start at a non-zero value of the bifurcation parameter.

### Time-dependent dynamics

In this section we analyze time-dependent dynamics in response to DNA damage. First, we consider the case in which DNA repair is suppressed; then, we consider the nominal model in which DNA repair mechanism is active; finally, we analyze how cell fate depends on the expression levels of Wip1 and PTEN.

#### Dynamics of the system with suppressed DNA repair

**Fig 6:**
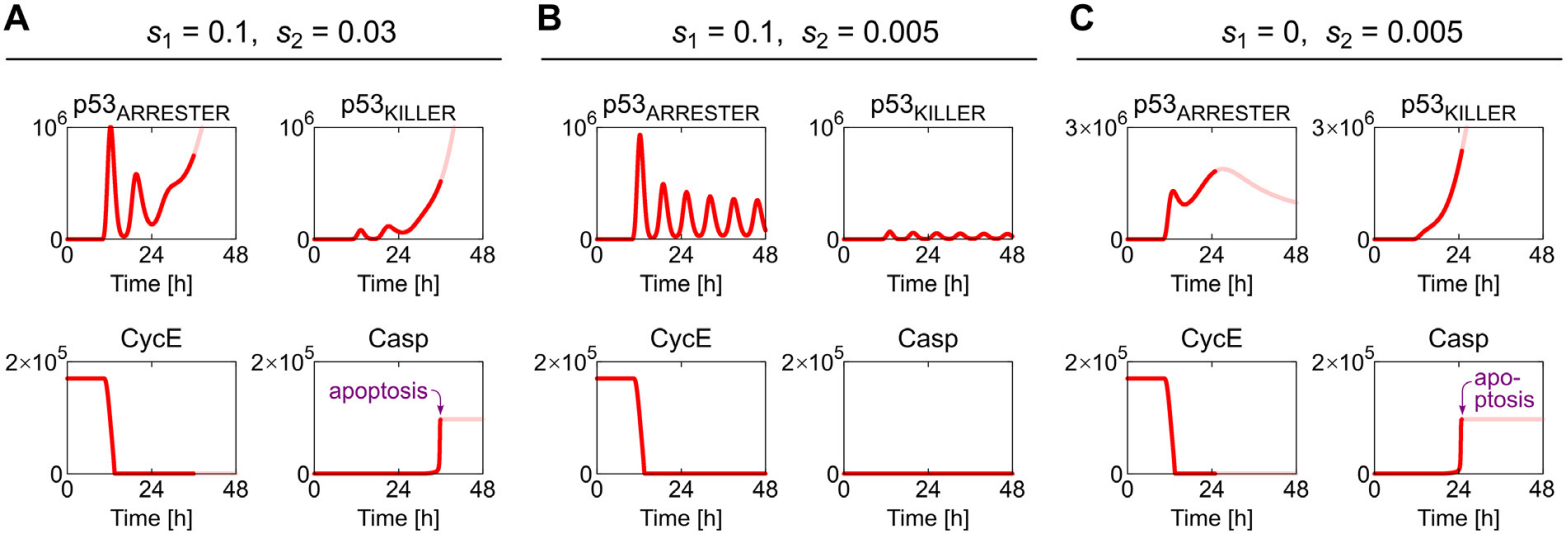
Deterministic simulation trajectories of the system with the suppressed DNA repair and either nominal or perturbed mRNA synthesis rates in response to 2-Gy irradiation. (A) Nominal Wip1 and PTEN mRNA synthesis rates (*s*_1_ = 0.1, *s*_2_ = 0.03). (B) Decreased PTEN mRNA synthesis rate (*s*_2_ = 0.005). (C) Decreased PTEN mRNA synthesis rate (*s*_2_ = 0.005) and zero Wip1 synthesis rate (*s*_1_ = 0). The simulation started at Time = 0 h from the steady state corresponding to the resting (unstimulated) cell. At Time = 10 h the irradiation phase started and lasted for 10 min. The faded line visualizes the trajectory after the initiation of apoptosis, and thus must be interpreted with caution.

In Fig 6 we analyze the system responses to the stimulation dose of 2 Gy, for nominal Wip1 and PTEN mRNA synthesis rates, i.e., s_1_ = 0.1, s_2_ = 0.03 (Fig 6A), as well as modified rates (Fig 6B and 6C). According to the bifurcation diagram (Fig 4) for the nominal parameters values, upon persistent DNA damage the system possesses the unique stable steady state. This state is reached after transient oscillations of p53_ARRESTER_ and p53_KILLER_ levels. Such response can be expected based on the bifurcation diagram shown in Fig 5A, indicating that the system generates oscillations for a sufficiently small PTEN synthesis rate, s_2_; here, the PTEN rate is larger but due to the slow accumulation of PTEN the system initially exhibits oscillations. Shortly after DNA is damaged, in the oscillatory phase, the level of p53_ARRESTER_ exceeds the cell cycle arrest threshold (see bifurcation point SN_1_ in Fig 2) leading to a rapid decrease of the free CycE level and suppression of the cell cycle. During this phase the p53_ARRESTER_ level remains below the apoptotic threshold (see the saddle—node bifurcations line in Fig 3B). After two oscillations the p53 killer grows to the high level, the apoptotic bifurcation line is surpassed and at about 25th hour since the DNA damage the cell is directed to apoptosis, a state characterized in the model by a high level of active caspases. Formally, within the model even in the apoptotic state the trajectory can still be calculated, but obviously initiation of apoptosis limits the validity of the model, and therefore after the apoptosis is initiated trajectories are drawn using faded lines.

In Fig 6B we analyze the response of the perturbed system in which PTEN synthesis rate, s_2_, is equal 0.005, i.e., is 6-fold lower than the nominal value, while Wip1 synthesis rate is at its nominal value (s_1_ = 0.1). For these parameters, as shown in the two-dimensional bifurcation diagram in Fig 4, the system possesses a unique stable recurrent solution, the limit cycle. Accordingly, as we see in Fig 6B, p53_ARRESTER_ and p53_KILLER_ exhibit sustained oscillations of period of about 6 hours. In these oscillations the level of p53_ARRESTER_ is high enough to suppress the cell cycle, while the amplitude of p53_KILLER_ oscillations does not exceed the apoptotic threshold. The observed behavior is similar to that exhibited by MCF-7 cells in the experiment of Geva-Zatorski et al. [73], in which irradiated cells showed quasiperiodic oscillation for 72 hours after irradiation. In these cells, the PTEN promoter is methylated and PTEN expression is not regulated by p53, and remains at a very low level.

In Fig 6C we analyze the response of the system with PTEN synthesis rate s_2_ = 0.005 and no Wip1 synthesis (s_1_ = 0). According to the two-dimensional bifurcation diagram in Fig 4 the system has the unique stable steady state. As shown in Fig 6C the level of p53_KILLER_ switches to the high level triggering apoptosis. In contrast to the case of nominal parameter values, the oscillatory phase is absent and the apoptosis is initiated considerably faster, about 15 h after DNA damage. The direct passage to the apoptotic state can be also deduced from Fig 4 showing that oscillations are not possible for s_1_ = 0, regardless of s_2_.

#### Responses of the intact system—dependence on the irradiation dose

**Fig 7:**
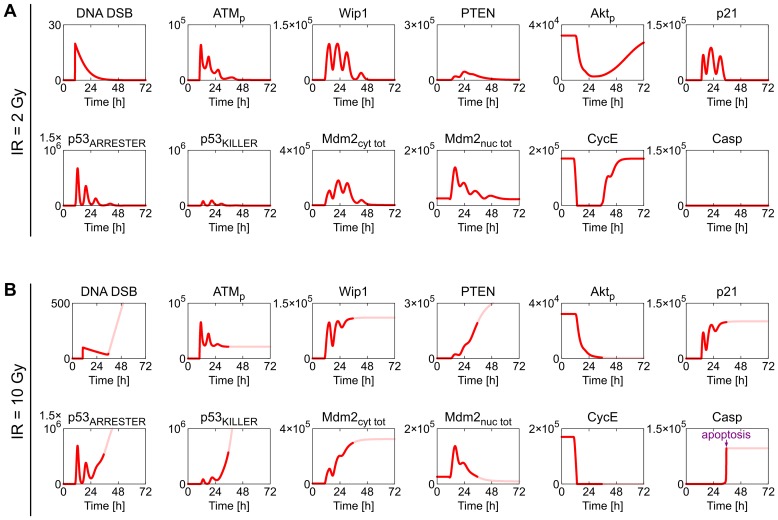
Deterministic simulation trajectories of the intact system in response to 2-Gy and 10-Gy irradiation. (A) 2-Gy irradiation is insufficient to trigger apoptosis and after several oscillations system recovers to the survival steady state. (B) 10-Gy irradiation is sufficient to trigger apoptosis: after two oscillations p53_KILLER_ stabilizes at a high level triggering apoptosis.

In Fig 7 we consider the model with nominal parameters and analyze the responses to two irradiation doses of 2 Gy and 10 Gy. As demonstrated in Fig 6A, in cells with suppressed DNA repair, the 2 Gy dose leads to apoptosis in about 25 hours since DNA damage. Here, the process is more complex as the repair of DNA may rescue cells from apoptosis. In this case the cell fate depends on the irradiation dose, and is decided through the competition between two processes: DNA repair and accumulation of PTEN which controls positive feedback-regulating levels of active Akt and p53_KILLER_. As shown in Fig 7A, small DNA damage resulting from 2-Gy irradiation can be almost fully repaired in about 20 hours, thus the apoptosis is not initiated. In contrast, the repair of extensive damage resulting from 10-Gy irradiation can be not accomplished sufficiently fast and the cell undergoes apoptosis. The time delay associated with PTEN accumulation serves as a clock, giving about 24 hours for DNA repair, and then directing cells with unrepaired DNA to apoptosis.

In Fig 7A we show trajectories of key model components after 2-Gy irradiation. DNA damage leads to a rapid ATM phosphorylation and activation. Activated ATM phosphorylates p53 at Ser15 and Ser20 to the p53_ARRESTER_ form, which accumulates and induces synthesis of p53 inhibitor, Mdm2, and phosphatase Wip1 which dephosphorylates ATM. These two negative feedback loops (mediated by Mdm2 and Wip1) lead to oscillations of the level of p53_ARRESTER_ and levels of p53_ARRESTER_-regulated proteins, including p21. Increased level of p21 leads to the inhibition of CycE and transient cell cycle arrest, during which DNA can be repaired.

p53_ARRESTER_ can be subsequently phosphorylated at Ser46 to the p53_KILLER_ form by HIPK2 (a kinase which accumulates in response to DNA damage; see detailed scheme of the model in S1 Fig). This process is opposed by Wip1 which converts p53_KILLER_ back to the p53_ARRESTER_ form. As a result, the level of p53_KILLER_ oscillates not surpassing the apoptotic threshold. After, about 20 h, when DNA repair is accomplished, ATM is deactivated and oscillations of p53_KILLER_ and p53_ARRESTER_ are terminated. Subsequently, the level of p21 drops below the threshold (bifurcation point SN_2_, Fig 2B), CycE returns to its initial level, and cell cycle progression is resumed.

In the case of extensive DNA damage caused by 10-Gy irradiation (Fig 7B) DNA repair requires more time. Although initial regulatory events are similar to those at 2-Gy irradiation, prolonged p53_KILLER_ activity induces accumulation of PTEN to a higher level, such that PTEN-mediated positive feedback loop terminates oscillations by sequestering Mdm2 in the cytoplasm, which separates it from its substrate, nuclear p53. Namely, accumulation of PTEN leads to dephosphorylation of Akt (see S1 Fig for details) and subsequent dephosphorylation of Mdm2 at Ser166 and Ser186, which blocks its nuclear entry and physically separates it from its target, p53. Of note, the positive feedback loop mediated by Wip1 (which stabilizes p53_ARRESTER_) and the one mediated by PTEN (which stabilizes p53_KILLER_) act antagonistically. In the case of extensive DNA damage, the slow PTEN-mediated positive feedback can ultimately take over, causing the surge of the level of p53_KILLER_ which ultimately surpasses the apoptosis-inducing threshold. Importantly, since early hours after DNA damage till (and throughout) apoptosis, the cell cycle remains suspended, preventing proliferation of cells which have lost their genomic integrity.

In summary, small DNA damage leads to a temporal cell cycle arrest during which DNA is repaired, while extensive DNA damage leads to the cell cycle arrest followed by apoptosis. Regulation of the core p53 module (see the bifurcation diagram in Fig 5) is such that in the oscillatory phase the level of p53_ARRESTER_ is high enough to suppress cell cycle but the level of p53_KILLER_ does not exceed the apoptotic threshold. Only in the “high” steady state levels of phosphorylated Akt and p53_KILLER_ exceed the apoptotic threshold. This type of regulation assures that the cell fate decision is unanimous.

#### Impact of Wip1 and PTEN expression levels on the sensitivity to irradiation

As shown in the previous subsection, in response to irradiation the cell can either undergo apoptosis or repair its DNA and survive. Cell fate decision depends on the irradiation dose as well as expression levels of regulatory proteins in the p53 pathway. Here, we analyze how Wip1 and PTEN mRNA synthesis rates s_1_ and s_2_, and the level of active PI3K influence the critical irradiation dose. Level of active PI3K depends on the total level of PI3K which is cell line-dependent and can increase in response to various stimuli including growth factors. Gene copy amplification and overexpression of PI3K have been found in several types of cancer, including gastric (SNU1), prostate (LNCaP), head and neck squamous cell carcinoma (HNSCC), ovarian (OVCAR4) and breast (MCF-12A, MB157) [63,64,66–68]. Wip1 and PTEN mRNA synthesis rates are also cell line-specific. In the lung (NCI-H522), breast (MCF-7, BT474, KPL-1, MDA-MB361), prostate (DU-145), colon (HCT15) and ovarian (OVCAR4) cancers, and in melanoma (UACC-257) and lymphoma (MOLT4), Wip1 expression is frequently elevated due to gene copy amplification (32); PTEN expression is decreased due to gene loss in colon (HCT116) and lung (NCI-H1299) cancers [87,88] or due to methylation of PTEN promoter in breast (MCF-7, BT-549) and non-small cell lung (A549) cancers [87,89].

**Fig 8:**
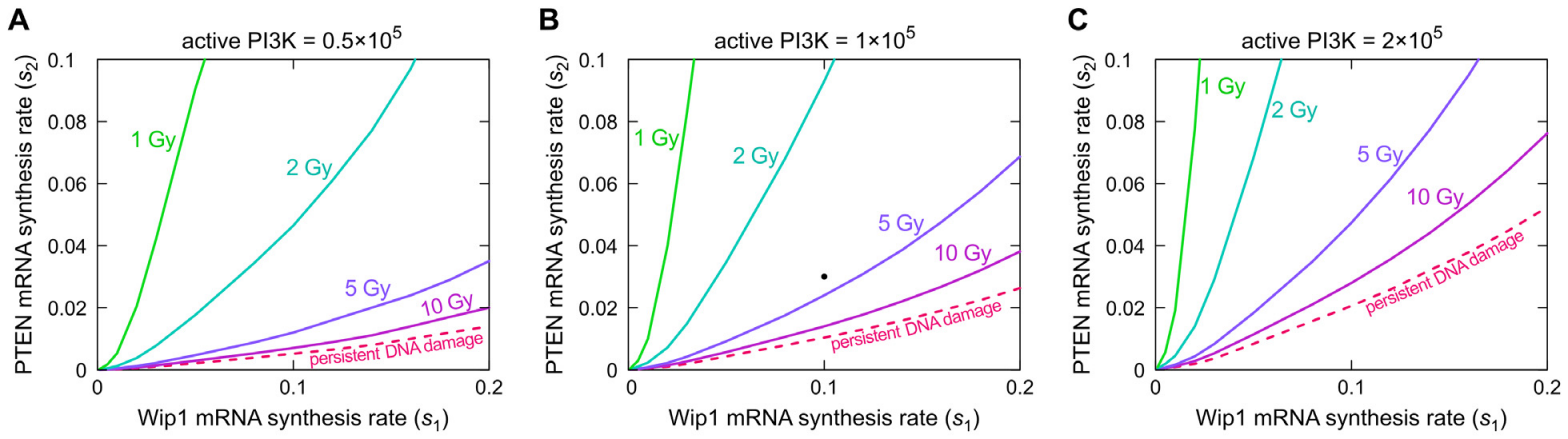
Critical irradiation doses as a function of Wip1 (*s*_1_) and PTEN (*s*_2_) mRNA synthesis rates. (A) Active PI3K level is decreased 2-fold from the nominal value. (B) The nominal value of active PI3K. (C) Active PI3K level is twice the nominal value. Color lines show the critical irradiation doses in the (*s*_1_, *s*_2_)-parameter space. Dashed line corresponds to the persistent (irreparable) DNA damage equal 100 DSBs. For each dose, the line separates the apoptotic region (above the line) and the survival region (below the line) in the (*s*_1_, *s*_2_)-parameter space. The black dot in (B) corresponds to nominal values of Wip1 and PTEN mRNA synthesis rates.

For nominal values of Wip1 and PTEN mRNA synthesis rates s_1_ and s_2_ assumed in the model and a nominal value of active PI3K the critical irradiation dose turns out to be 4.05 Gy. In Fig 8 we show the apoptotic and survival regions in the (s_1_, s_2_) parameter plane for four irradiation doses: 1 Gy, 2 Gy, 5 Gy, 10 Gy, as well as the persistent (irreparable) DNA damage (equal 100 DSBs). We consider three levels of active PI3K: nominal, twofold decreased, and twofold increased. For each level of active PI3K the apoptotic region (above separatrix) increases and the survival region (below separatrix) shrinks with the irradiation dose. Interestingly, even for irreparable DNA damage cells can survive provided that the ratio s_2_/s_1_ (PTEN to Wip1) is sufficiently low, as in the case analyzed in Fig 6B. The critical irradiation dose increases with increasing Wip1 and decreases with increasing PTEN. The ratio s_2_/s_1_ (PTEN to Wip1 mRNA synthesis rate) is roughly constant on each of survival/apoptosis separatrices (and increases with the irradiation dose) conforming the antagonistic relationship between Wip1 and PTEN.

One may notice that the ratio s_2_/s_1_ for each of the separatrices increases with the PI3K level (compare Fig 8A, 8B and 8C). This is, for a given Wip1 mRNA synthesis rate an increase of PTEN synthesis rate can be compensated by an increase of active PI3K. This relationship follows from the fact that PTEN and PI3K has directly opposing roles in the pathway.

Overall, our analysis indicates that cellular proclivity for irradiation-induced apoptosis increases with PTEN to Wip1 expression ratio, and decreases with the level of active PI3K. This confirms the pro-survival role of Wip1 and PI3K, and the pro-apoptotic role of PTEN. Since cancer cell lines have diverse expression levels of Wip1, PTEN and PI3K, they are expected to exhibit divergent responses to irradiation; therefore, different cancers may have widely varying sensitivity to radiotherapy. In particular, the performed analysis explains high survival rates of irradiated MCF-7 cells for which the expression of Wip1 is significantly increased while PTEN expression is reduced. This cell line (studied by Geva-Zatorski et al. [73]) respond to high irradiation dose by long-lasting oscillations, showing that even the persistent DNA damage does not induce apoptosis.

### The role of stochasticity

**Fig 9:**
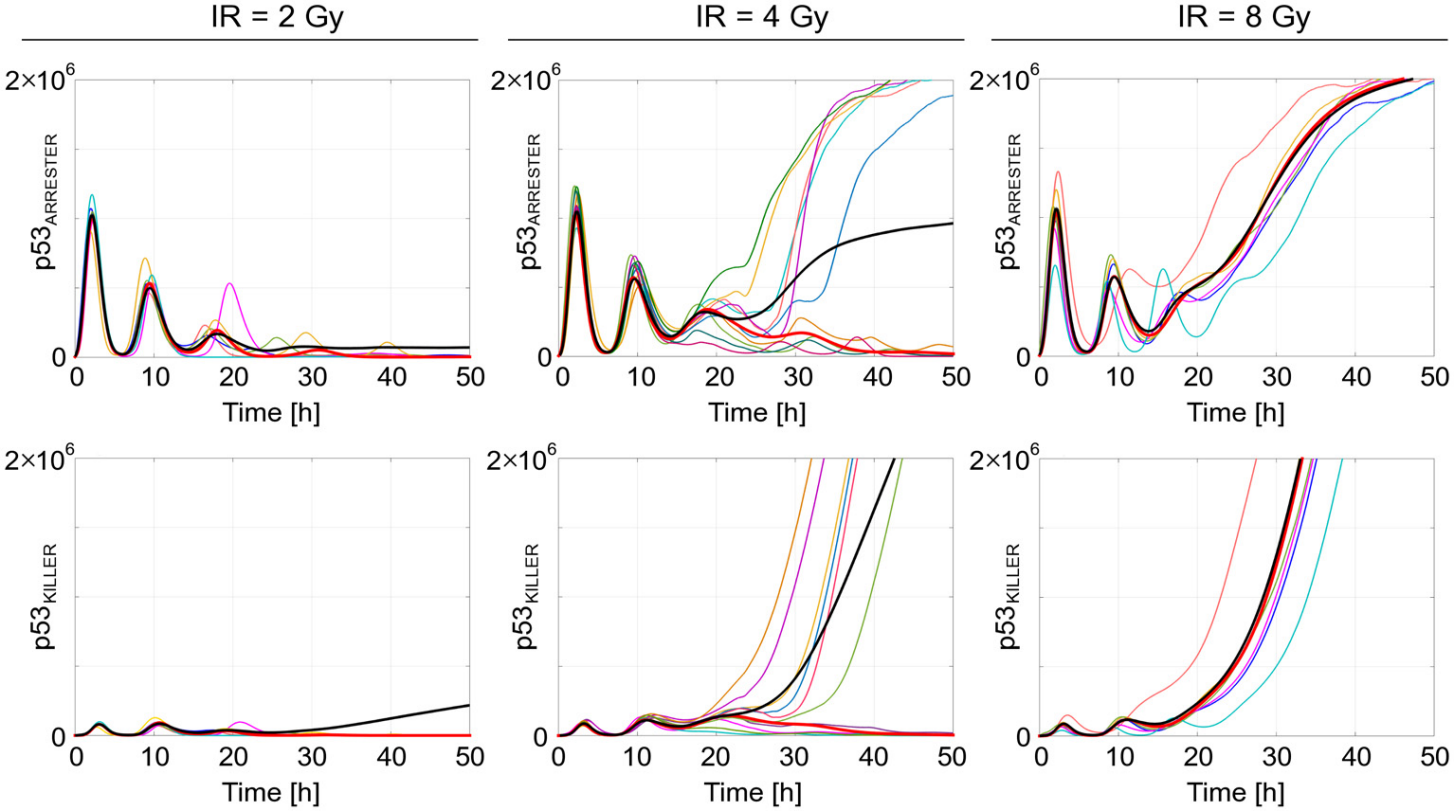
Stochastic vs. deterministic simulation trajectories in the response to low (2 Gy), intermediate (4 Gy) and high (8 Gy) irradiation doses. Single-cell stochastic trajectories—thin color lines; average over 1000 stochastic trajectories—bold black line; deterministic approximation—bold red line.

In order to analyze the cell-to-cell variability we performed stochastic simulations according to the Gillespie algorithm [90] implemented in BioNetGen [91]. In Fig 9 we compare trajectories obtained in single-cell stochastic simulations with the deterministic trajectory as well as the population trajectory, i.e., trajectory obtained by averaging over 1000 single-cell stochastic trajectories. In the figure we show trajectories of p53_ARRESTER_ and p53_KILLER_ in response to three irradiation doses: small of 2 Gy, intermediate of 4 Gy (which is just below the critical dose for the deterministic approximation) and large of 8 Gy. As one can observe, the stochastic trajectories follow closely the deterministic trajectory for the low and high doses, and in these two cases the population trajectory almost matches the deterministic one. This is in contrast to the intermediate dose of 4 Gy, for which the deterministic trajectory after a few oscillations (during which DNA is repaired) returns to the initial state characterized by low levels of p53_ARRESTER_ and p53_KILLER_, while the stochastic trajectories separate into two groups corresponding to the apoptotic cells (of high p53 levels) and surviving cells (of low p53 levels). As a result, for 4 Gy the deterministic trajectory is much different from the population-averaged trajectory. Such discrepancy between deterministic and stochastic solutions has been demonstrated earlier for various, even very simple, nonlinear or multistable systems [92–97]. It is especially pronounced in the cases of bistable (or multistable) systems when the deterministic trajectory converges to one of steady states, while the stochastic trajectories split into two (or more) groups. In this case the population average (observed in population experiments by Western blotting or PCR) does not correspond to any single-cell trajectory.

**Fig 10:**
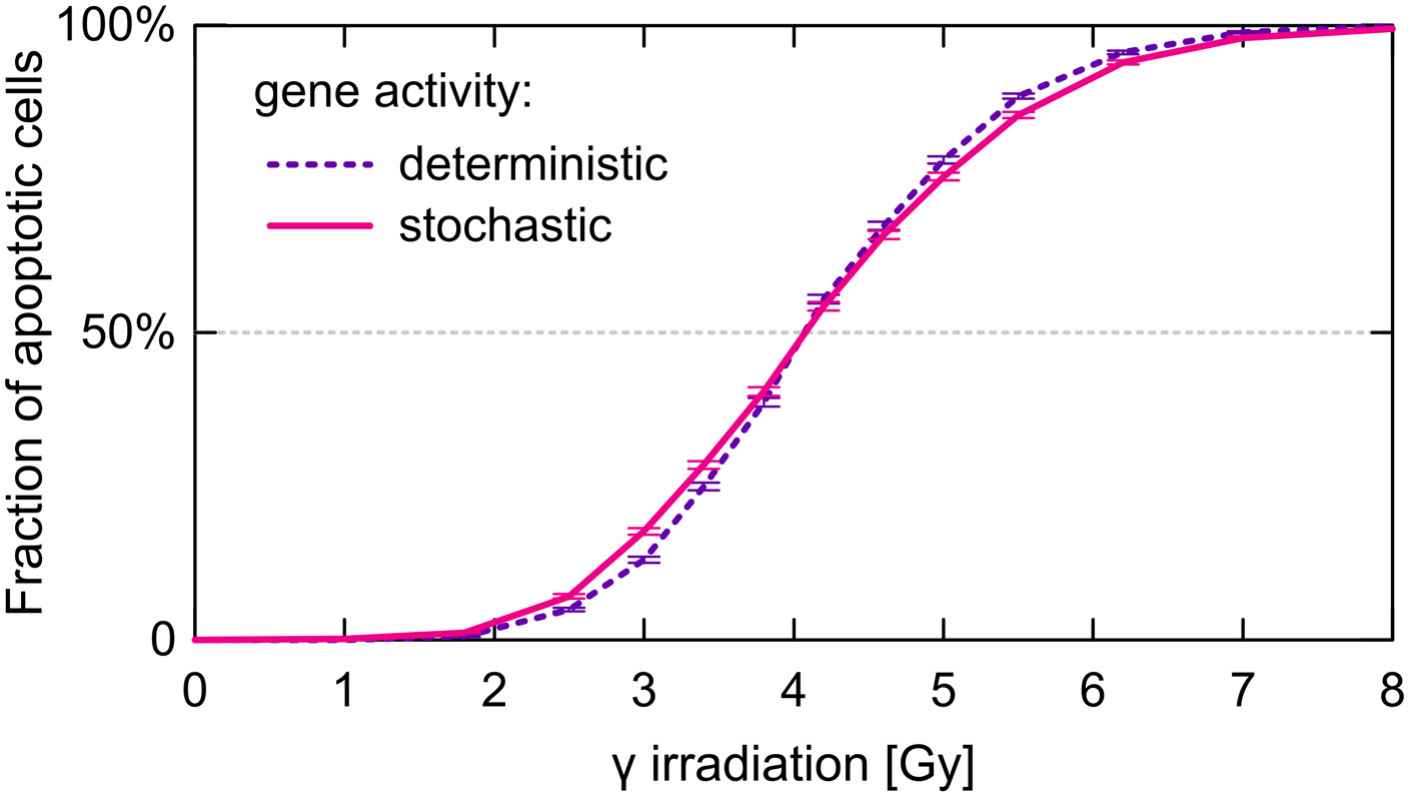
Fraction of apoptotic cells as a function of irradiation dose for two types of stochastic simulations. For each of analyzed doses the fraction and corresponding error was calculated based on 5000 stochastic single-cell simulations. The state of the cell was checked at 72. hour after irradiation which, as shown in Fig 11, is sufficient for unanimous cell fate decision.

The fraction of apoptotic cells increases with the irradiation dose (see Fig 10, where 5000 single-cell stochastic simulations were performed for each of 14 different doses from the 0–8 Gy range). For the critical (deterministic) dose equal 4.05 Gy, the fraction of apoptotic cells was found to be close to 50%. The fraction of apoptotic cells increases from about 10% to about 90% as the irradiation dose increases from 2.5 Gy to 6 Gy. In order to determine how gene switching noise contributes to the heterogeneity in cell fate decisions we performed simulations in a model variant in which gene activity was assumed to be a deterministic function of the level of p53 (in a gene-specific phosphoform). As expected, after removing one source of stochasticity the individual cell responses has become less heterogeneous (see also S4 Fig), which is manifested by the steeper sigmoid of the fraction of apoptotic cells (violet vs. pink line). Surprisingly, the difference between these two cases is not very large, which indicates that transcriptional and translational noises significantly contribute to the overall stochasticity.

**Fig 11:**
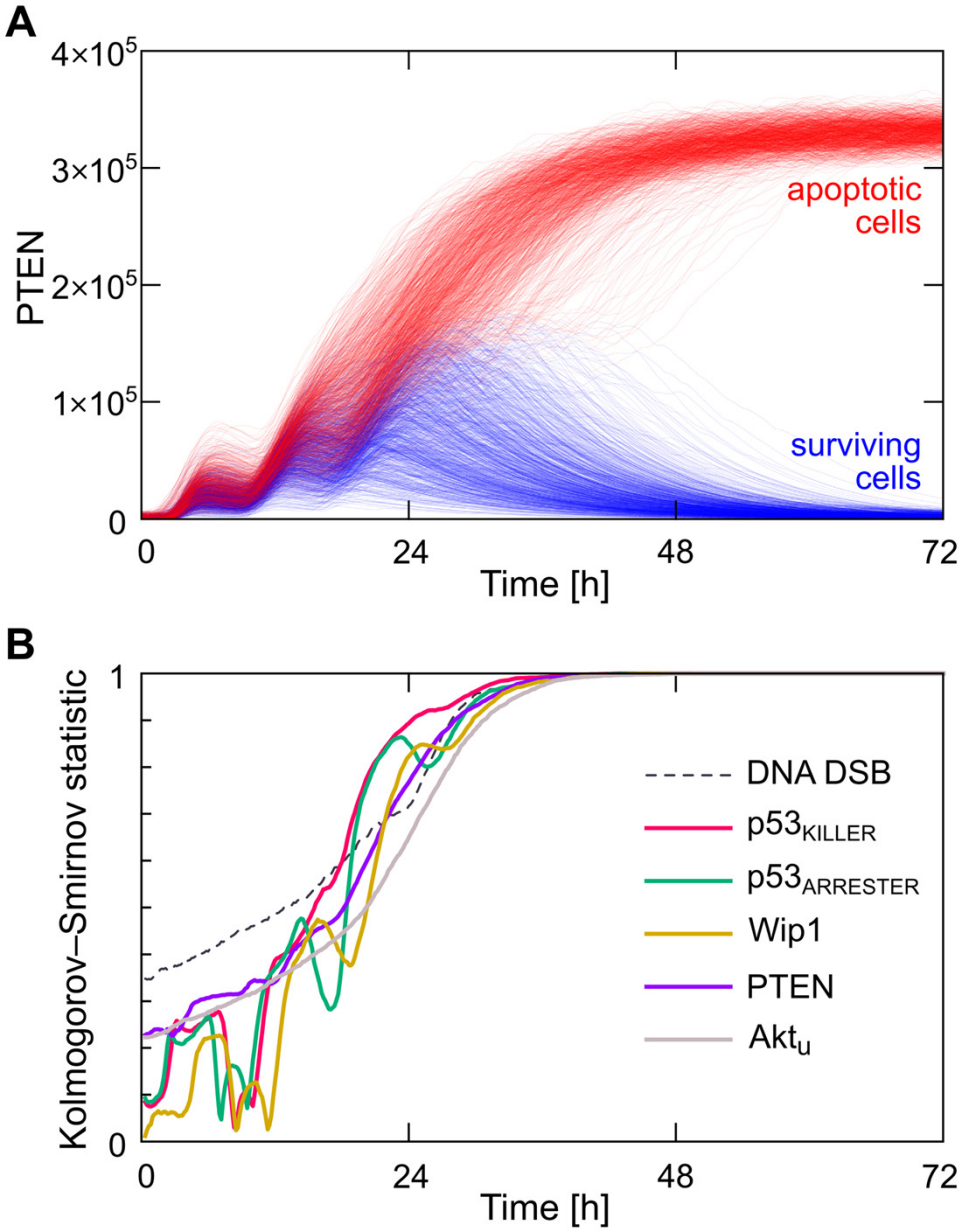
Analysis of the cell fate decision-making process. (A) PTEN levels in subpopulations of surviving (blue) and apoptotic (red) cells after 4-Gy irradiation. 10 000 cells are simulated in total. (B) Kolmogorov—Smirnov statistics between distributions of six variables which characterize surviving and apoptotic cells.

As showed above for the intermediate doses, the cell population splits into apoptotic and surviving subpopulations. In Fig 11 we analyze how the cell fate decision after 4-Gy irradiation is reached in time. In Fig 11A we show trajectories of PTEN, which is a key protein mediating slow positive feedback loop and thus its level was found to be a good predictor of the ultimate cell fate decision. As shown, the trajectories of surviving cells (blue) are almost fully separated from trajectories of apoptotic cells (red) after about 36 hours. The separation between apoptotic and surviving trajectories progresses in time, as shown in Fig 11B where temporal evolution of the Kolmogorov—Smirnov statistic between 6 distributions of different variables characterizing apoptotic and surviving cells is calculated. This figure confirms that the cell fate decision is reached after about 36 hours, as for this time Kolmogorov—Smirnov statistics (for all variables) reaches 1, which indicates that distributions corresponding to apoptotic and surviving cells fully separate. The number of DSBs that arise during the irradiation phase (which, due to the stochasticity, can be different for cells irradiated with the same dose) is the fastest predictor. PTEN and Akt_u_ (levels of which can be different prior to the irradiation due to stochastic fluctuations in resting cells) are also fast predictors and, moreover, the Kolmogorov—Smirnov statistics corresponding to their distributions grow monotonically. This is in contrast to p53_KILLER_, p53_ARRESTER_, and Wip1 levels, which exhibit pronounced oscillations after irradiation, and for which the Kolmogorov—Smirnov statistics reach 1 non-monotonously.

## Discussion

p53 is thoroughly studied as a pivotal signaling node that integrates and transforms diverse stress signals into downstream responses including cell cycle arrest and apoptosis to limit proliferation of cells with the damaged genetic material. Based on the experimental knowledge about feedbacks and interactions within the p53 network, we proposed a modular model of p53 signaling pathway, in which the dynamics of the core p53 module controls downstream modules that govern cell cycle arrest and apoptosis. The model has the purposeful bifurcation structure that delineates plausible temporal responses and allows for, we think, the most unambiguous cell death, cycle arrest, or survival decisions.

The stochastic, yet unambiguous, processing of analog irradiation signals to reach a digital cell fate outcome is assured by the bistability of cell cycle arrest and apoptotic modules, but primarily by a specific-type bistability of the core p53 module. As demonstrated by the two-parameter bifurcation analysis of the core module, in a relatively broad range of parameters the limit cycle oscillations (of p53_ARRESTER_ and p53_KILLER_ levels) coexist with the stable steady state in which the p53_KILLER_ level is very high, while the level of phosphorylated Akt is very low. The limit cycle oscillations of p53_ARRESTER_ induce cell cycle arrest, while the high p53_KILLER_ level and low level of phosphorylated Akt induce apoptosis. The key difference between dynamics of the cycle arrest module and the apoptotic module is that the former is based on reversible bistability and involves one input signal, p53_ARRESTER_, which suppresses cell cycle when above the threshold and releases the cell cycle arrest when below the lower threshold. In turn, apoptosis induction requires two signals (increase of p53_KILLER_ and decrease of phosphorylated Akt) simultaneously, and is an irreversible process.

The bifurcation structure delineates temporal responses to DNA damage. Small DNA damage results in temporal cell cycle arrest, followed by DNA repair and return to the resting cell state, while high DNA damage results in the cell cycle arrest and subsequent apoptosis (without returning to cell cycle). The direct jump between limit cycle oscillations and the stable steady state is enabled by the presence of a subcritical Neimark—Sacker bifurcation in which the limit cycle loses its stability merging with an unstable invariant torus. Interestingly, this type of bifurcation, rarely analyzed in the context of molecular regulatory networks, can exist in at least three-dimensional systems. Although the core module is 18-dimensional, the stability analysis of the steady state showed that at most three eigenvalues have positive real parts which implies that locally the system could be reduced to a three-dimensional one. It remains an open question whether such reduction is possible globally.

We propose that in the process of cell fate decision-making the p53 system can exhibit the most robust transitions when the only stable recurrent solutions are a stable limit cycle and a stable steady state (out of this limit cycle), provided there exists a parameter region for which these recurrent solutions coexist. Such structure allows cells to switch to the cell cycle arrest (associated in the model with limit-cycle oscillations of the levels of p53 phosphoforms) and then to direct transition to either apoptosis or recovery. Interestingly, there exists an even simpler bifurcation structure, shown in S5 Fig, that allows for such transitions. In this hypothetical case, as the bifurcation parameter s_2_ increases the limit cycle simply vanishes in the saddle—loop bifurcation. The most pronounced dynamical difference between the modeled and the hypothetical scenario is that the period of oscillations diverges to infinity as the bifurcation parameter approaches the saddle—loop bifurcation point. This would imply that in a heterogeneous cellular population some cells would exhibit much longer oscillation periods, which is not observed in experiments [73]. As discussed by us earlier [76], we thus expect that oscillations in the p53 system (as well as in the NF-κB system) do not arise or vanish through a saddle—loop bifurcation or a saddle—node on invariant cycle bifurcation (also called SNIPER) in which limit cycle arises from orbit homoclinic to the saddle.

Biochemical signal processing depends on the topology and time scales of feedback loops. Regulatory networks with negative feedback(s) can produce oscillatory or pulsed responses to tonic stimuli, and allow for adaptation to the change in the level of stimulation. The existence of positive feedbacks, in turn, can give rise to bistability [98] (or multistability [99]) that can be harnessed for cell fate decisions. Combinations of positive and negative feedbacks allow for more complex behaviors. At least three qualitatively different type of responses leading to distinct regulatory outcomes are possible. (1) In the case when a positive feedback loop acts on a short time scale and is embedded in a longer negative feedback loop, the system can switch periodically between two predefined states (e.g., the autophagy/translation switch [100]). (2) In the case when a positive feedback loops encompasses the negative one, the system can exhibit autonomous oscillations (e.g., oscillations in the TNF—NF-κB autocrine system [101]). (3) In the case when a slow positive feedback loop can turn off a negative feedback, the system can exhibit biphasic responses in which after an oscillatory phase it either returns to the initial state (more likely when the signal is short-lasting) or it switches to another steady state (more likely when the signal is persistent or lasts sufficiently long [102]). The last case (which is a simplification of the p53 regulatory core organization discussed in this study) seems to be a good candidate for the unbiased and proportionate process of cell fate decisions. The oscillatory phase provides a time interval to collect and integrate various signals, and estimate the severity of stress, before a (potentially irreversible) switch to another committed state is done.

Stochastic simulations of the model allowed to analyze how the death-or-life decision is achieved in time, and what are the best or earliest indicators of apoptosis. By calculating time-dependent Kolmogorov—Smirnov statistic between distributions of protein levels corresponding to two populations of cells with apoptotic and surviving fates, we found that the clear separation between these two populations is reached at about 30 hours after irradiation. PTEN, the p53-induced phosphatase that mediates slow positive feedback loop, was found to be the most robust and fast apoptosis predictor. Interestingly, the Kolmogorov—Smirnov statistics for Wip1, p53_ARRESTER_ and p53_KILLER_ are non-monotonous in time, what possibly follows from the pronounced oscillations of these components and indicates that at some time-points of the response the elevated or decreased levels of pro- or anti-apoptotic proteins may not correlate with the ultimate cell fate decision.

Most of experimental data were gathered for cancer (or immortalized) cell lines, which became cancerous as a result of malfunctions in the p53 network. Much less is known about regulation of normal, i.e., non-cancer, non-immortalized cells. Nevertheless, we expect that the plausible responses of normal cells should consist of the cell cycle suppression phase (which can be associated with p53 oscillations), followed by either apoptosis or recovery, depending on the extent of the DNA damage. Accordingly, in contrast to the majority of previous studies focused on modeling of cancer cell lines, we aimed at constructing the model that reflects behavior of normal cells after γ irradiation. Within this model, the responses of different cancer cell lines can be analyzed by adjusting the appropriate parameters such as mRNA synthesis rates or steady-state levels of proteins that are assumed to be not regulated in the model. In this way one can simulate cancer cell lines that are known to have elevated expression of Wip1 such as MCF-7, BT474 (breast cancers), OVCAR4 (ovarian cancer), MOLT4 (lymphoma), U2OS (osteosarcoma) [50–52,54,103], lines of decreased PTEN expression such as MCF-7 (breast cancer), H1299, H322, Calu1 (lung cancers) [58–60], or with amplification of PI3K gene, such as MKN1, SNU1 (gastric cancers), OVCAR4, A2780 (ovarian cancers) [63–70].

The life-or-death decision is reached in the interplay of two antagonistic phosphatases Wip1 and PTEN. Our analysis shows that sensitivity to apoptosis increases with PTEN expression and decreases with Wip1 or PI3K expression, which confirms the pro-survival functions of Wip1 and PI3K, and pro-apoptotic functions of PTEN. The other key regulatory pro-apoptotic protein present in network is kinase HIPK2 that converts p53_ARRESTER_ to the p53_KILLER_ phosphoform [104]. The model shows that undamped oscillations of p53_ARRESTER_ and p53_KILLER_ levels observed in MCF-7 cells can result from the low expression of PTEN. Analogously, our analysis indicates that cell lines with Wip1 overexpression (resulting from, e.g., gene amplification) can exhibit persistent oscillations and resistance to apoptosis. Strong dependence of the critical irradiation dose on PTEN, Wip1, and PI3K expression may suggest that pretreatment of cancer cells (with intact p53) with drugs that act temporally to elevate the expression of PTEN or to repress the expression of Wip1 or PI3K can enhance the effectiveness of radiotherapy.

**Fig 12:**
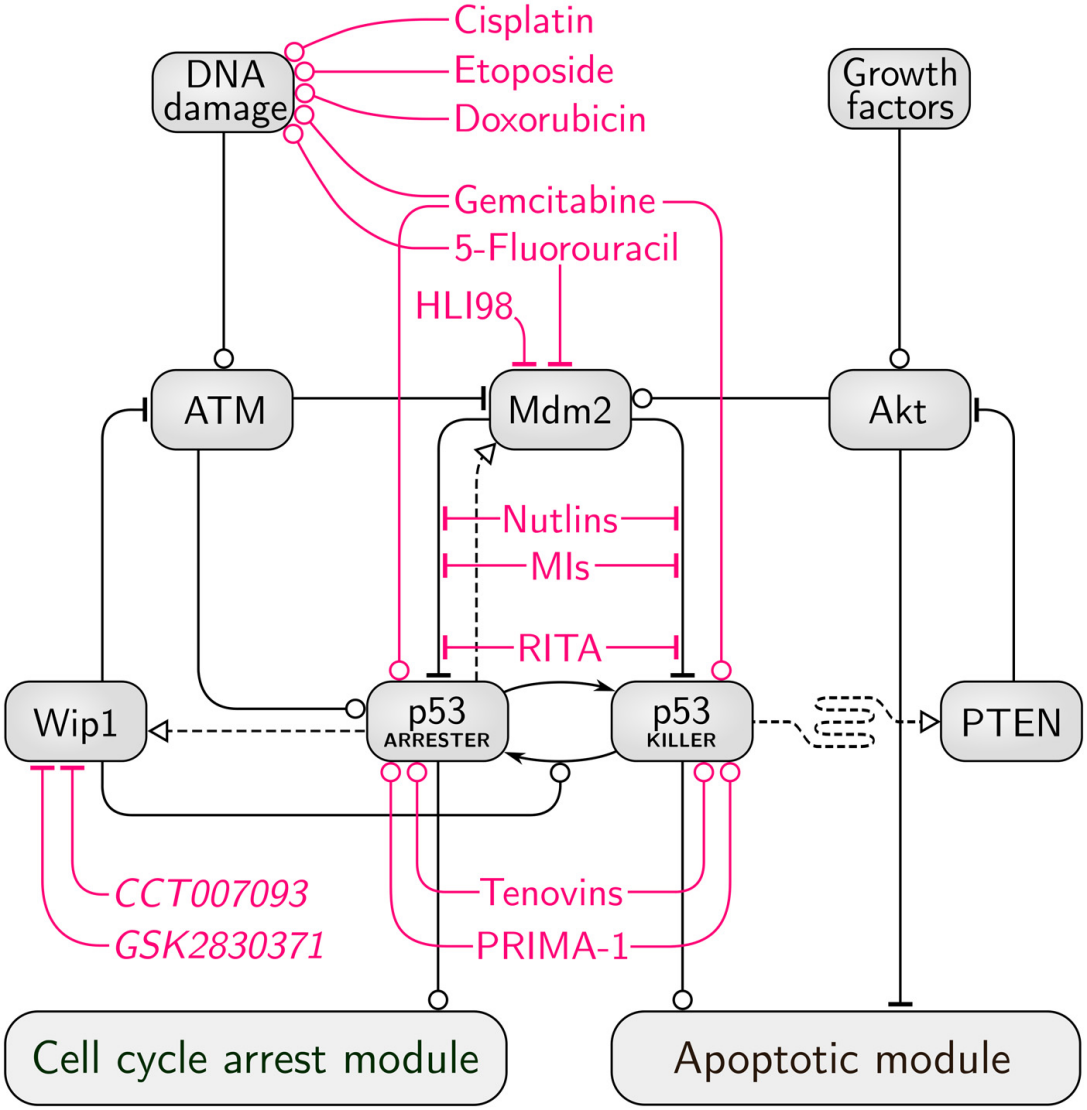
Chemotherapeutic agents targeting the p53 regulatory core. A selection of chemical compounds, both clinically approved chemotherapeutic drugs and newly discovered inhibitors, are shown. Some agents have pleiotropic effects (gemcitabine, 5-fluoroacil); several p53 inhibitors may be effective only towards a mutated, conformationally disrupted p53 (PRIMA-1 and similar not shown); tenovins have activating influence on p53 because they inhibit p53 inhibitor, sirtuin. Nutlins, MIs and RITA inhibit Mdm2 and p53 interaction.

Very recently, Puszyński et al. [105] have modified his earlier model [80] to study the responses of the p53 network to nutlins, promising antitumor chemical agents which bind to a p53-binding pocket of Mdm2 and thus hinder Mdm2 from ubiquitinating p53. Puszyński et al. demonstrated *in silico* that dose-splitting can be ineffective at low doses but effective at high doses, which can be attributed to nonlinear behavior of the regulatory system, manifested by the fact that a certain p53 threshold has to be exceeded to induce apoptosis. We propose that the current model can be used to study combination therapies involving agents which reduce the expression or inhibit the activity of Wip1, Mdm2, PI3K, together with ionizing radiation. In Fig 12 we survey over agents known to inhibit the selected nodes of the p53 pathway as well as DNA-damaging compounds that can be used in place of irradiation [106,107]. The proposed model provides the opportunity to investigate responses of particular cancer types, for which the anomalies in expression of p53 inhibitors are characterized. The aim is to propose a treatment that would reduce the levels or activity of Wip1, Mdm2, PI3K in cancer cells to make them more sensitive to radiotherapy, and to devise optimal drug and irradiation protocols that would leverage the impact of inhibitors by synchronizing their administration with the induced DNA damage.

The regulatory proteins Wip1, PTEN, PI3K, and HIPK2 present in the model are themselves important nodes of a larger regulatory network thus their levels and activity can be modulated by numerous other proteins or stimuli. For example, Wip1 expression is upregulated by not only p53 but also c-Jun, nuclear factor κB (NF-κB), cyclic adenosine monophosphate response element-binding protein (CREB), E2F transcription factor 1 (E2F1), Estrogen Receptor-alpha (ERα) [108–112]. PTEN expression is upregulated by early growth-response protein 1 (EGR1), or downregulated by Proto-Oncogene Polycomb Ring Finger (BMI1), NF-κB, c-Jun, Snail Family Zinc Finger 1 (SNAI1), oncogenic factor inhibitor of DNA binding 1 (ID1), ecotropic virus integration site 1 protein (EVI1) [113–116]. Neurogenic locus notch homolog protein 1 (NOTCH1) can either upregulate PTEN through CBF1 (C-repeat binding factor 1) or downregulate it through V-Myc Avian Myelocytomatosis Viral Oncogene Homolog (Myc) [115]. PI3K is activated by various growth and survival factors, including fibroblast growth factor (FGF), vascular endothelial cell growth factor (VEGF), human growth factor (HGF), angiopoietin I (Ang1), insulin, receptor tyrosine kinases (RTKs), G-protein-coupled receptors (GPCRs) [40,117]. These and other existing connections allow to expand the *in silico* drug impact analysis onto a larger network, and dissect possible dynamical consequences of inhibition of proteins more distant from p53 in the network.

## Materials and Methods

### Stochastic and deterministic model representations

The ordinary differential equations were integrated numerically in Matlab. Stochastic simulations were performed using the Gillespie algorithm implemented in BioNetGen. Bifurcation diagrams were obtained with Matcont. The Matlab code (full model, core module, cell cycle arrest module, apoptotic module), BioNetGen code (full model) and Matlab/Matcont files (core module, cell cycle arrest module, apoptotic module) are provided as S1, S2 and S3 Codes.

### Stochastic simulations

To account for initial heterogeneity in protein levels in cell population the 10-min long irradiation phase was preceded by a 100-hour long simulation of resting cells, except for S4 Fig, where the resting phase lasted for 300 hours. Average protein levels in Fig 9 were obtained by averaging over 1000 independent stochastic simulations; the fraction of apoptotic cells with respect to the dose was determined based on 5000 stochastic simulations; the (time-dependent) two-sample Kolmogorov—Smirnov statistic between apoptotic and surviving cell subpopulations was calculated based on 10 000 stochastic simulations, Fig 11.

The stratification into surviving and apoptotic cells was based on the level of active caspases at 72. hour after the irradiation phase; cells with the level of active caspases higher (lower) than the threshold defined as the value of active caspases at the saddle-node bifurcation point (SN in Fig 3C) were considered apoptotic (surviving). The Kolmogorov—Smirnov statistic is defined as *D*_a,s_(*t*) = sup_*x*_|*F*_a_(*x*,*t*) − *F*_s_(*x*,*t*)| where *F*_a_ and *F*_s_ are time-dependent cumulative distributions for the surviving and apoptotic subpopulations, calculated numerically, and sup is the supremum function.

## Supporting Information

S1 Text This supplementary document includes: overview of mathematical models of the p53 system, detailed description of the model, three tables containing the notation guide, list of parameters, and list of reactions. (PDF)

S1 Fig Detailed representation of the full model. Arrow-headed dashed lines indicate transcriptional regulation, arrow-headed solid lines—protein transformation, circle-headed solid lines—positive influence, hammer-headed dotted lines—ubiquitination by Mdm2 leading to protein degradation. The subscripts *n* or *c* denote either nuclear or cytoplasmic localization of Mdm2. Bold ‘P’ and non-bold ‘U’ denote phosphorylated and unphosphorylated states of given residues, respectively. Pro-survival and cycle-promoting proteins are represented with blue boxes, pro-apoptotic proteins with yellow boxes, proteins involved in cell cycle arrest with green boxes, while the remaining proteins and protein complexes are left in grey boxes.
(PDF)

S2 Fig The negative feedback mediated by Wip1 is required for generating oscillations. Trajectories for p53 and Mdm2 were simulated using the model variant in which there is no Wip1-mediated dephosphorylation of ATM. Simulations were performed for four different pairs of Wip1 (*s*_1_) and PTEN (*s*_2_) synthesis rates, with IR dose equal 3 Gy. Black line corresponds to nominal values of *s*_1_ and *s*_2_.
(PDF)

S3 Fig Recurrent solutions for p53_KILLER_ as a function of Wip1 synthesis rate, active PI3K level and DNA damage level. PTEN mRNA synthesis rate is equal to the nominal value s_2_ = 0.03; Wip1 synthesis rate is equal s_1_ = 0.2 in (B) and s_1_ = 0.1 in (C). The number of DSBs is equal 100 for (A) and (B). The stable and unstable steady states are indicated by solid and dashed lines, respectively. Dots and open circles show the maxima and minima of the stable and unstable limit cycles, respectively. Green vertical line shows the Neimark—Sacker bifurcation (N—S). Red dots mark saddle—node bifurcations (SN_1_, SN_2_), yellow dots mark the supercritical Hopf (H_super_) and the subcritical Hopf (H_sub_) bifurcation. Note the log-scale on the vertical axis. The bifurcation diagrams with respect to Wip1 and PI3K resemble the mirror image of the bifurcation diagram with respect to PTEN (see main text Fig 5A). The bifurcation diagram with respect to DNA damage is similar to the bifurcation diagram with respect to PTEN, but the limit cycle oscillations start at non-zero value of the bifurcation parameter.
(PDF)

S4 Fig Influence of the gene switching rates on the single-cell stochastic trajectories for p53_tot_ and Mdm2_tot_. (A) and (B): nominal gene switching rates. (C) and (D): 10-fold increased gene switching rates. The irradiation phase started at Time = 300 h and lasted for 10 min, the irradiation dose was 4 Gy. The dynamics of the p53_tot_ and Mdm2_tot_ weakly depends on the gene switching rate, although its increase leads to some decrease of the amplitude of fluctuations in unstimulated cells. Notice the logarithmic scale on vertical axes.
(PDF)

S5 Fig A hypothetical alternative bifurcation structure for the core module. The stable limit cycle (of envelope marked by pairs of blue points) coexists with a high-level stable steady state and disappears through the saddle—loop bifurcation (SL). The stable and unstable steady states are indicated by solid and dashed lines, respectively; red dots labeled SN mark saddle—node bifurcations.
(PDF)

S1 Code ZIP-archived directory containing Matlab scripts and a ReadMe file. (ZIP)

S2 Code ZIP-archived directory containing a BioNetGen model file and a ReadMe file. (ZIP)

S3 Code ZIP-archived directory containing Matlab scripts calling Matcont functions, and a ReadMe file. (ZIP)
