# Supplementary material for: Feedbacks, Bifurcations, and Cell Fate Decision-Making in the p53 System
Source: PLoS Comput Biol. 2016 Feb 29;12(2):e1004787. doi: 10.1371/journal.pcbi.1004787 (PMC4771203; doi:10.1371/journal.pcbi.1004787)
Supplement: S1 Text — (PDF) [file pcbi.1004787.s001.pdf]

Supporting Information – S1 Text
(with Tables A, B, C)

featuring the article

Feedbacks, Bifurcations, and Cell Fate Decision-Making in the p53 System

by Beata Hat, Marek Kochańczyk, Marta N. Bogdał, and Tomasz Lipniacki

PLOS Computational Biology 2016

Overview of mathematical models of the p53 system

Generation of oscillations

Bar-Or et al. [S1] found that oscillations arise due to the existence of the negative feedback loop coupling p53 with its inhibitor Mdm2 and proposed a three-component model with a hypothetical intermediate which introduces time delay between accumulation of p53 and accumulation of its inhibitor Mdm2, explaining the observed damped oscillations. Later, when Lahav et al. [S2] and Geva-Zatorski et al. [S3] demonstrated experimentally that single cells can exhibit undamped oscillations of p53 and Mdm2 levels, Ma et al. [S4] attributed time delays to the processes of Mdm2 transcription and translation, which resulted in the model featuring limit cycle oscillations. Later, Batchelor et al. [S5] demonstrated experimentally that oscillations require recurrent initiation of ATM pulses by Wip1 and proposed a model based on two negative feedback loops: one coupling p53 with Mdm2, the other involving p53, ATM, Chk2, and Wip1.

In the theoretical study from Tyson group, Ciliberto et al. [S6] introduced a positive feedback (in which p53 inhibits indirectly nuclear translocation of Mdm2, while Mdm2 degrades p53 in the nucleus) and demonstrated that it leads to more robust oscillations arising with non-zero amplitude either in the saddle–node–loop (SNL, also known as saddle node on invariant circle, SNIC) or in the cyclic fold bifurcation accompanying the subcritical Hopf bifurcation. However, the problem with SNL bifurcation is that in this bifurcation oscillations arise with infinite period while the period of oscillations in cells seems to be similar and roughly conserved, see Hat et al. [S7] for discussion.

p53 and cell fate decisions

Wee and Aguda (2006) [S8] demonstrated that the presence of the positive feedback following the scheme p53 → PTEN –| Pip3 → Akt → Mdm2 –| p53 introduces bistability that can be harnessed to control cell fate decisions.

Tyson group, extending their previous work [S6], proposed a small three-component model in which the positive feedback arises from the assumption that p53 synthesis is positively regulated by cytoplasmic Mdm2 [S9]. This model exhibits limit cycle oscillation between two cyclic fold bifurcations associated with subcritical Hopf bifurcations. The model was combined with the apoptotic/cell cycle arrest model in which three forms of p53 where introduced: p53-killer (that activates apoptotic genes like PUMA, p53DINP1 and p53AIP1), p53-helper (that induces p21 and Wip1 production, blocks CDK activity) and p53-lurker (that induces p21 production). In the resulting model, pulses of p53 lead to cell cycle arrest and, if sustained, to cell death.

Later, we (Puszynski et al. 2008 [S10]) proposed a more complex model of p53 regulation, exhibiting both oscillations and bistability. We found that the intact p53 system can exhibit oscillations in response to DNA damage, which can be either terminated when DNA is repaired, or the system may switch to the apoptotic state of a high p53 level when DNA repair is not accomplished in sufficiently short time. The positive feedback loop considered earlier by Wee and Aguda (2006) [S8] allows for switching to the apoptotic state and works as a clock. The cell can return to homeostasis if DNA repair is accomplished before the signal is relayed through the PTEN-controlled loop which inhibits Mdm2. We demonstrated that PTEN-deficient cells (such as MCF-7 line cells) exhibit sustained oscillations without triggering apoptosis. The idea was explored later by Wee et al. (2009) [S11], who augmented the p53 regulatory core with an apoptotic module involving Bax, Bad, Bcl-2, and Bcl-x_L.

Dynamics very similar to that of [S10] was achieved in an elaborate model proposed by Zhang et al. (2011) [S12]. The important modification introduced by the group of Zhang (see also Zhang et al. (2009) [S13] and Zhang et al. (2010) [S14]) was the inclusion of distinct phosphorylation states of p53: $p53_{ARRESTER}$ and $p53_{KILLER}$ which regulate different groups of genes. The model of Zhang et al. [S12] encompasses also the negative feedback loop mediated by Wip1, introduced earlier by Batchelor et al. [S5]. This allowed to analyze the competition between the p53/PTEN/Akt/Mdm2 and the ATM/p53/Wip1 feedbacks during DNA repair, and attribute pro- and anti-apoptotic roles to PTEN and Wip1, respectively.

The model

The proposed model of the p53 regulatory network consists of three modules: p53 core, cell cycle arrest module and apoptotic module. The cell cycle arrest and apoptotic modules have been described in detail in the main text. The detailed scheme of the core module is presented in Figure S1. Here, we describe briefly considered interactions and provide references to the literature.

DNA damage leads to the activation of ATM by phosphorylation at Ser1981 [S15,S16]. Activated ATM phosphorylates p53 at Ser15 and Ser20 to the $p53_{ARRESTER}$ form leading to its transcriptional activation and stabilization (reduction of the degradation rate) [S17–S23]. Simultaneously, ATM phosphorylates p53 inhibitor Mdm2 at Ser395 leading to its inactivation and destabilization (increase of the degradation rate) [S24]. Additionally, ATM phosphorylates SIAH1 at Ser19 leading to disruption of the HIPK2–SIAH1 complex resulting in HIPK2 accumulation [S25]. Kinase HIPK2 phosphorylates $p53_{ARRESTER}$ at Ser46 to the $p53_{KILLER}$ form [S26–S29]. $p53_{ARRESTER}$ and $p53_{KILLER}$ have different target genes. $p53_{ARRESTER}$ induces synthesis of p53 inhibitor Mdm2 [S30,S31], anti-apoptotic phosphatase Wip1 [S32] and cell cycle suppressor p21 [S33]. In turn, $p53_{KILLER}$ induces synthesis of pro-apoptotic protein Bax [S34] and pro-apoptotic phosphatase PTEN [S35].

Wip1 has 3 targets in the model; It dephosphorylates: ATM at Ser1981 [S36], Mdm2 at Ser395 (leading to its stabilization) [S37], and $p53_{KILLER}$ at Ser46 to $p53_{ARRESTER}$. PTEN mediates long positive feedback loop that stabilizes p53: it dephosphorylates PIP3 to PIP2 while PIP3 enables membrane localization of pro-survival kinase Akt, allowing its activation via phosphorylation at Thr308 [S38–S40]. Activated Akt phosphorylates Mdm2 at Ser166 and Ser186 enabling its translocation to the nucleus [S41], where it ubiquitinates all forms of p53 promoting their degradation by the proteasome [S42,S43]. This way PTEN accumulation leads to inhibition of Akt [S39], which itself is the activator of p53 inhibitor Mdm2 [S43]. Action of PTEN is opposed by growth factor stimulation leading to activation of kinase PI3K that phosphorylates PIP2 to PIP3 [S44,S45].

There are three outcomes from the core module, p21, Bax, and phosphorylated Akt. p21 regulates cell cycle arrest module in such a way that increase of p21 above some threshold leads suppression of cell cycle, while decrease of p21 below some lower threshold allows cell to return to the cycle. Bax and Akt regulate apoptotic module, in such a way that simultaneous increase of Bax level and decrease of phosphorylated Akt level lead to the irreversible apoptosis.

Supporting Tables

Table A. Notation guide.

Symbol	Description
	Core module
DNA_{DSB}	DNA damage due to IR: double strand breaks (DSBs)
ATM	kinase ATM
ATM_p	ATM phosphorylated at Ser1981 (upon DNA DSBs)
$Wip1_{gene}$	state of the Wip1 gene: active/inactive
$Wip1_{mRNA}$	Wip1 transcript
Wip1	phosphatase Wip1
$SIAH1_u$	unphosphorylated SIAH1
$SIAH1_p$	SIAH1 phosphorylated at Ser19
HIPK2	kinase HIPK2
$p53_{0p}$	unphosphorylated p53
$p53_{ARRESTER}$	p53 phosphorylated at Ser15, Ser20
$p53_{KILLER}$	p53 phosphorylated at Ser15, Ser20 and additionally at Ser46
$p53_{s46}$	p53 phosphorylated at Ser46 only
$Mdm2_{gene}$	state of the Mdm2 gene: active/inactive
$Mdm2_{mRNA}$	Mdm2 transcript
$Mdm2_{cyt\_0p}$	cytoplasmic, unphosphorylated Mdm2
$Mdm2_{cyt\_2p}$	cytoplasmic Mdm2 phosphorylated at Ser166 and Ser186
$Mdm2_{nuc\_2p}$	nuclear Mdm2 phosphorylated at Ser166 and Ser186
$Mdm2_{nuc\_3p}$	nuclear Mdm2 phosphorylated at Ser166, 186 and additionally at Ser395
PI3K	kinase PI3K
$PTEN_{gene}$	state of the PTEN gene: active/inactive
$PTEN_{mRNA}$	PTEN transcript
PIP2	bi-phosphatidylinositol
PIP3	tri-phosphatidylinositol
Akt_u	unphosphorylated AKT
Akt_p	Akt phosphorylated at Thr308
	Apoptotic module
Bax_{gene}	state of the Bax gene: active/inactive
Bax_{mRNA}	Bax transcript
Bax	unbound form of Bax
$Bclx_L$	unbound form of Bcl-x_L
Bax : $Bclx_L$	complex of Bax and Bcl-x_L
Bad_u	unbound, unphosphorylated Bad
Bad_p	Bad: unbound, phosphorylated at Ser75 and Ser99
$Bclx_L$: Bad_u	complex of Bcl-x_L and Bad_u
14-3-3	unbound adapter protein 14-3-3
Bad_p : 14-3-3	complex of Bad_p and 14-3-3
proCasp	inactive caspase
Casp	active caspase
	Cell cycle arrest module
$p21_{gene}$	state of the p21 gene: active/inactive
$p21_{mRNA}$	p21 transcript
p21	unbound p21
CycE	unbound Cyclin E
p21: CycE	complex of p21 and Cyclin E
$Rb1_u$	Rb1: unbound, unphosphorylated at Ser780
$Rb1_p$	Rb1: unbound, phosphorylated at Ser780
$Rb1_u$: E2F1	complex of unphosphorylated Rb1 and E2F1

Table B. List of parameters.

Parameter	Symbol	Value	Remarks	Ref.
Duration of the IR phase	IR_{T}	600 [s]	—	this study
IR dose	IR_{Gy}	1,2,3,4,10 [Gy]	—	this study
Number of DSBs per 1Gy of IR	DSB_{Gy}	10	—	[S4]*
Maximal number of DSBs	DSB_{max}	10^6	—	[S4]
Number of repair complexes	DSB_{rep}	20	—	[S4]
Total amount of Rb1	$Rb1_{\mathrm{tot}}$	3×10^5 [mlcs/cell]	$Rb1_{\mathrm{tot}} = Rb1_{\mathrm{p}}(t) + Rb1_{\mathrm{u}}(t) + Rb1_{\mathrm{u}}{:}E2F1(t)$	this study
Total amount of E2F1	$E2F1_{\mathrm{tot}}$	2×10^5 [mlcs/cell]	$E2F1_{\mathrm{tot}} = E2F1(t) + Rb1_{\mathrm{u}}{:}E2F1(t)$	this study
Total amount of Akt	Akt_{tot}	10^5 [mlcs/cell]	$Akt_{\mathrm{tot}} = Akt_{\mathrm{u}}(t) + Akt_{\mathrm{p}}(t)$	this study
Total amount of PIP3 and PIP2	PIP_{tot}	10^5 [mlcs/cell]	$PIP_{\mathrm{tot}} = PIP2(t) + PIP3(t)$	this study

*We consider only DSBs that undergo slow repair.

Table C. List of reactions.

Reaction	Rate	Coeff(s)	Value
Core module			
$\varnothing \xrightarrow{\mathrm{IR}} \mathrm{DNA_{DSB}}$	$h_1 \cdot \dfrac{DSB_{\mathrm{Gy}} \cdot IR_{\mathrm{Gy}}}{IR_{\mathrm{T}}} \cdot (DSB_{\mathrm{max}} - DNA_{\mathrm{DSB}})$	h_1<br>DSB_{Gy}<br>IR_{Gy}<br>IR_T<br>DSB_{max}<br>h_2	10^{-6}<br>10<br>1, 2, 3, 4, 10<br>600<br>10^6<br>10^{-13}
$\varnothing \xrightarrow{\mathrm{Casp}} \mathrm{DNA_{DSB}}$	$h_2 \cdot Casp \cdot (DSB_{\mathrm{max}} - DNA_{\mathrm{DSB}})$		
$\mathrm{DNA_{DSB}} \rightarrow \varnothing$	$\dfrac{rep}{DNA_{\mathrm{DSB}} + DSB_{\mathrm{rep}}}$	rep<br>DSB_{rep}	10^{-3}<br>20
$\mathrm{ATM} \xrightarrow{\mathrm{DNA_{DSB}}} \mathrm{ATM_p}$	$p_1 \cdot \dfrac{DNA^h_{\mathrm{DSB}}}{M^h_1 + DNA^h_{\mathrm{DSB}}}$	p_1<br>h<br>M_1	3×10^{-4}<br>2<br>5
$\mathrm{ATM} \xleftarrow{\mathrm{Wip1}} \mathrm{ATM_p}$	$d_1 \cdot Wip1$	d_1	10^{-8}
$\mathrm{SIAH\text{-}1} \xrightarrow{\mathrm{ATM_p}} \mathrm{SIAH\text{-}1_p}$	$p_2 \cdot ATM_{\mathrm{p}}$	p_2	10^{-8}
$\mathrm{SIAH\text{-}1} \leftarrow \mathrm{SIAH\text{-}1_p}$	d_2	d_2	3×10^{-5}

$\varnothing \rightarrow \mathrm{HIPK2}$	s_8	s_8	3×10^{-5}
$\mathrm{HIPK2} \xrightarrow{\mathrm{Mdm2_{nuc\_2p}},\ \mathrm{SIAH1}} \varnothing$	$g_7 \cdot (SIAH1_{\mathrm{u}} + Mdm2_{\mathrm{nuc\_2p}})^2$	g_7	3×10^{-5}
$\varnothing \xrightarrow{\mathrm{p53_{KILLER}}} \mathrm{Wip1_{mRNA}}$	$s_1 \cdot \dfrac{q_{0\_\mathrm{Wip1}} + q_{1\_\mathrm{Wip1}} \cdot p53^h_{\mathrm{KILLER}}}{q_2 + q_{0\_\mathrm{Wip1}} + q_{1\_\mathrm{Wip1}} \cdot p53^h_{\mathrm{KILLER}}}$	s_1<br>q_{0_Wip1}<br>q_{1_Wip1}<br>h<br>q_2	0.1<br>10^{-5}<br>3×10^{-13}<br>2<br>3×10^{-3}
$\mathrm{Wip1_{mRNA}} \rightarrow \varnothing$	g_1	g_1	3×10^{-4}
$\varnothing \rightarrow \mathrm{Wip1}$	$t_1 \cdot Wip1_{\mathrm{mRNA}}$	t_1	3×10^{-5}
$\mathrm{Wip1} \rightarrow \varnothing$	g_8	g_8	3×10^{-13}
$\varnothing \rightarrow \mathrm{p53_{0p}}$	s_6	s_6	300
$\mathrm{p53_{0p}} \rightarrow \varnothing$	g_{101}	g_{101}	0.1×10^{-13}
$\mathrm{p53_{0p}} \xrightarrow{\mathrm{Mdm2_{nuc\_2p}}} \varnothing$	$g_{11} \cdot Mdm2^2_{\mathrm{nuc\_2p}}$	g_{11}	100×10^{-13}
$\mathrm{p53_{ARRESTER}} \xrightarrow{\mathrm{Mdm2_{nuc\_2p}}} \varnothing$<br>$\mathrm{p53_{KILLER}} \xrightarrow{\mathrm{Mdm2_{nuc\_2p}}} \varnothing$<br>$\mathrm{p53_{s46}} \xrightarrow{\mathrm{Mdm2_{nuc\_2p}}} \varnothing$	$g_{12} \cdot Mdm2^2_{\mathrm{nuc\_2p}}$	g_{12}	10^{-13}
$\mathrm{p53_{0p}} \xrightarrow{\mathrm{ATM_p}} \mathrm{p53_{ARRESTER}}$	$p_3 \cdot ATM_{\mathrm{p}}$	p_3	3×10^{-8}
$\mathrm{p53_{0p}} \leftarrow \mathrm{p53_{ARRESTER}}$	d_3	d_3	10^{-4}
$\mathrm{p53_{0p}} \xrightarrow{\mathrm{HIPK2}} \mathrm{p53_{s46}}$	$p_4 \cdot HIPK2$	p_4	10^{-10}
$\mathrm{p53_{0p}} \leftarrow \mathrm{p53_{s46}}$	$d_4 \cdot Wip1$	d_4	10^{-10}
$\varnothing \xrightarrow{\mathrm{p53_{ARRESTER}}} \mathrm{Mdm2_{mRNA}}$	$s_3 \cdot \dfrac{q_{0\_\mathrm{Mdm2}} + q_{1\_\mathrm{Mdm2}} \cdot p53^h_{\mathrm{ARRESTER}}}{q_2 + q_{0\_\mathrm{Mdm2}} + q_{1\_\mathrm{Mdm2}} \cdot p53^h_{\mathrm{ARRESTER}}}$	s_3<br>q_{0_Mdm2}<br>q_{1_Mdm2}<br>h<br>q_2	0.1<br>10^{-4}<br>3×10^{-13}<br>2<br>3×10^{-3}
$\mathrm{Mdm2_{mRNA}} \rightarrow \varnothing$	g_1	g_1	3×10^{-4}
$\mathrm{Mdm2_{mRNA}} \rightarrow \mathrm{Mdm2_{cyt\_0p}}$	$t_3 \cdot Mdm2_{\mathrm{mRNA}}$	t_3	0.1
$\mathrm{Mdm2_{cyt\_0p}} \xrightarrow{\mathrm{AKT_p}} \mathrm{Mdm2_{cyt\_2p}}$	$p_5 \cdot Akt_{\mathrm{p}}$	p_5	10^{-8}
$\mathrm{Mdm2_{cyt\_0p}} \leftarrow \mathrm{Mdm2_{cyt\_2p}}$	d_5	d_5	10^{-4}
$\mathrm{Mdm2_{cyt\_2p}} \rightarrow \mathrm{Mdm2_{nuc\_2p}}$	i_1	i_1	10^{-3}
$\mathrm{Mdm2_{nuc\_2p}} \xrightarrow{\mathrm{ATM_p}} \mathrm{Mdm2_{nuc\_3p}}$	$p_6 \cdot ATM_{\mathrm{p}}$	p_6	10^{-8}
$\mathrm{Mdm2_{nuc\_2p}} \xleftarrow{\mathrm{Wip1}} \mathrm{Mdm2_{nuc\_3p}}$	$d_6 \cdot Wip1$	d_6	10^{-10}
$\mathrm{Mdm2_{cyt\_0p}} \rightarrow \varnothing$	g_{14}	g_{14}	10^{-13}
$\mathrm{Mdm2_{cyt\_2p}} \rightarrow \varnothing$<br>$\mathrm{Mdm2_{nuc\_2p}} \rightarrow \varnothing$	g_{15}	g_{15}	3×10^{-14}
$\mathrm{Mdm2_{nuc\_3p}} \rightarrow \varnothing$	g_{16}	g_{16}	10^{-13}
$\varnothing \xrightarrow{\mathrm{p53_{KILLER}}} \mathrm{PTEN_{mRNA}}$	$s_2 \cdot \dfrac{q_{0\_\mathrm{PTEN}} + q_{1\_\mathrm{PTEN}} \cdot p53^h_{\mathrm{KILLER}}}{q_2 + q_{0\_\mathrm{PTEN}} + q_{1\_\mathrm{PTEN}} \cdot p53^h_{\mathrm{KILLER}}}$	s_2<br>q_{0_PTEN}<br>q_{1_PTEN}<br>h<br>q_2	0.03<br>10^{-5}<br>3×10^{-13}<br>2<br>3×10^{-3}
$\mathrm{PTEN_{mRNA}} \rightarrow \varnothing$	g_2	g_2	3×10^{-4}
$\varnothing \rightarrow \mathrm{PTEN}$	$t_2 \cdot PTEN_{\mathrm{mRNA}}$	t_2	0.1
$\mathrm{PTEN} \rightarrow \varnothing$	g_6	g_6	10^{-13}
$\mathrm{PIP2} \xrightarrow{\mathrm{PI3K}} \mathrm{PIP3}$	$p_8 \cdot PI3K$	p_8	3×10^{-9}
$\mathrm{PIP2} \xleftarrow{\mathrm{PTEN}} \mathrm{PIP3}$	$d_7 \cdot PTEN$	d_7	3×10^{-7}
$\mathrm{Akt} \xrightarrow{\mathrm{PIP3}} \mathrm{Akt_p}$	$p_{12} \cdot PIP3$	p_{12}	10^{-9}
$\mathrm{Akt} \leftarrow \mathrm{Akt_p}$	d_8	d_8	10^{-4}

Apoptotic module			
$\varnothing \xrightarrow{\text{p53}_{\text{KILLER}}} \text{Bax}_{\text{mRNA}}$	$s_4 \cdot \dfrac{q_{0\_\text{Bax}} + q_{1\_\text{Bax}} \cdot p53^h_{\text{KILLER}}}{q_2 + q_{0\_\text{Bax}} + q_{1\_\text{Bax}} \cdot p53^h_{\text{KILLLER}}}$	s_4<br>q_{0_Bax}<br>q_{1_Bax}<br>h<br>q_2	0.03<br>10^{-5}<br>3×10^{-13}<br>2<br>3×10^{-3}
$\text{Bax}_{\text{mRNA}} \rightarrow \varnothing$	g_4	g_4	3×10^{-4}
$\varnothing \rightarrow \text{Bax}$	$t_4 \cdot Bax_{\text{mRNA}}$	t_4	0.1
$\text{Bax} \rightarrow \varnothing$	g_9	g_9	10^{-13}
$\text{Bax} \rightarrow \text{Bclx}_{\text{L}}$	b_1	b_1	3×10^{-5}
$\text{Bax} \leftarrow \text{Bclx}_{\text{L}}$	u_1	u_1	10^{-3}
$\text{Bax:Bclx}_{\text{L}} \rightarrow \text{Bclx}_{\text{L}}$	g_{16}	g_{16}	10^{-13}
$\text{Bclx}_{\text{L}} + \text{Bad}_{\text{u}} \rightarrow \text{Bclx}_{\text{L}}\text{:Bad}_{\text{u}}$	b_2	b_2	3×10^{-3}
$\text{Bclx}_{\text{L}} + \text{Bad}_{\text{u}} \leftarrow \text{Bclx}_{\text{L}}\text{:Bad}_{\text{u}}$	u_2	u_2	10^{-3}
$\text{Bclx}_{\text{L}}\text{:Bad}_{\text{u}} \xrightarrow{\text{Akt}_{\text{p}}} \text{Bclx}_{\text{L}}$	$p_7 \cdot Akt_{\text{p}}$	p_7	3×10^{-9}
$\text{Bad}_{\text{u}} \xrightarrow{\text{AKT}_{\text{p}}} \text{Bad}_{\text{p}}$	$p_7 \cdot Akt_{\text{p}}$	p_7	3×10^{-9}
$\text{Bad}_{\text{u}} \leftarrow \text{Bad}_{\text{p}}$	d_9	d_9	3×10^{-5}
$\text{Bad}_{\text{p}} + \text{14-3-3} \rightarrow \text{Bad}_{\text{p}}\text{: 14-3-3}$	b_3	b_3	3×10^{-3}
$\text{Bad}_{\text{p}} + \text{14-3-3} \leftarrow \text{Bad}_{\text{p}}\text{: 14-3-3}$	u_3	u_3	10^{-3}
$\text{Bad}_{\text{p}}\text{: 14-3-3} \rightarrow \text{Bad}_{\text{u}} + \text{14-3-3}$	d_9	d_9	3×10^{-5}
$\varnothing \rightarrow \text{proCasp}$	s_7	s_7	30
$\text{proCasp} \xrightarrow{\text{Bax, Casp}} \text{Casp}$	$a_1 \cdot Bax + a_2 \cdot Casp^2$	a_1<br>a_2	3×10^{-10}<br>10^{-12}
$\text{proCasp} \rightarrow \varnothing$<br>$\text{Casp} \rightarrow \varnothing$	g_{17}	g_{17}	3×10^{-13}
Cell cycle arrest module			
$\varnothing \xrightarrow{\text{p53}_{\text{ARRESTER}}} \text{p21}_{\text{mRNA}}$	$s_5 \cdot \dfrac{q_{0\_p21} + q_{1\_p21} \cdot p53^h_{\text{ARRESTER}}}{q_2 + q_{0\_p21} + q_{1\_p21} \cdot p53^h_{\text{ARRESTER}}}$	s_5<br>q_{0_p21}<br>q_{1_p21}<br>h<br>q_2	0.1<br>10^{-5}<br>10^{-13}<br>2<br>3×10^{-3}
$\text{p21}_{\text{mRNA}} \rightarrow \varnothing$	g_5	g_5	3×10^{-4}
$\varnothing \rightarrow \text{p21}$	$t_5 \cdot p21_{\text{mRNA}}$	t_5	0.1
$\text{p21} \rightarrow \varnothing$	g_{19}	g_{19}	3×10^{-13}
$\text{p21} + \text{CycE} \rightarrow \text{p21:CycE}$	b_5	b_5	10^{-5}
$\text{p21} + \text{CycE} \leftarrow \text{p21:CycE}$	u_6	u_6	10^{-14}
$\text{p21:CycE} \rightarrow \varnothing$	g_{20}	g_{20}	10^{-13}
$\text{Rb1} \xrightarrow{\text{CycE}} \text{Rb1}_{\text{p}}$	$p_9 \cdot CycE$	p_9	3×10^{-6}
$\text{Rb1} \leftarrow \text{Rb1}_{\text{p}}$	$\dfrac{d_{12}}{M_2 + Rb1_{\text{p}}}$	d_{12}<br>M_2	10^4<br>10^5
$\text{Rb1}_{\text{u}} + \text{E2F1} \rightarrow \text{Rb1}_{\text{u}}\text{: E2F1}$	b_4	b_4	10^{-5}
$\text{Rb1}_{\text{u}} + \text{E2F1} \leftarrow \text{Rb1}_{\text{u}}\text{: E2F1}$	u_5	u_5	10^{-14}
$\text{Rb1}_{\text{u}}\text{: E2F1} \xrightarrow{\text{CycE}} \text{Rb1}_{\text{p}} + \text{E2F1}$	$p_{10} \cdot CycE$	p_{10}	3×10^{-6}

Supplementary references

S1. Bar-Or RL, Maya R, Segel LA, Alon U, Levine AJ, Oren M. Generation of oscillations by the p53-Mdm2 feedback loop: A theoretical and experimental study. *Proc Natl Acad Sci USA*. 2000;97: 11250–11255. doi:10.1073/pnas.210171597

S2. Lahav G, Rosenfeld N, Sigal A, Geva-Zatorsky N, Levine AJ, Elowitz MB, et al. Dynamics of the p53-Mdm2 feedback loop in individual cells. *Nat Genet*. 2004;36: 147–150. doi:10.1038/ng1293

S3. Geva-Zatorsky N, Rosenfeld N, Itzkovitz S, Milo R, Sigal A, Dekel E, et al. Oscillations and variability in the p53 system. *Mol Syst Biol*. 2006;2: 2006.0033. doi:10.1038/msb4100068

S4. Ma L, Wagner J, Rice JJ, Hu W, Levine AJ, Stolovitzky GA. A plausible model for the digital response of p53 to DNA damage. *Proc Natl Acad Sci USA*. 2005;102: 14266–14271. doi:10.1073/pnas.0501352102

S5. Batchelor E, Mock CS, Bhan I, Loewer A, Lahav G. Recurrent initiation: a mechanism for triggering p53 pulses in response to DNA damage. *Mol Cell*. 2008;30: 277–289. doi:10.1016/j.molcel.2008.03.016

S6. Ciliberto A, Novak B, Tyson JJ. Steady states and oscillations in the p53/Mdm2 network. *Cell Cycle*. 2005;4: 488–493.

S7. Hat B, Puszynski K, Lipniacki T. Exploring mechanisms of oscillations in p53 and nuclear factor-B systems. *IET Syst Biol*. 2009;3: 342–355. doi:10.1049/iet-syb.2008.0156

S8. Wee KB, Aguda BD. Akt versus p53 in a Network of Oncogenes and Tumor Suppressor Genes Regulating Cell Survival and Death. *Biophys J*. 2006;91: 857–865. doi:10.1529/biophysj.105.077693

S9. Zhang T, Brazhnik P, Tyson JJ. Exploring mechanisms of the DNA-damage response: p53 pulses and their possible relevance to apoptosis. *Cell Cycle*. 2007;6: 85–94.

S10. Puszyński K, Hat B, Lipniacki T. Oscillations and bistability in the stochastic model of p53 regulation. *J Theor Biol*. 2008;254: 452–465. doi:10.1016/j.jtbi.2008.05.039

S11. Wee KB, Surana U, Aguda BD. Oscillations of the p53-Akt Network: Implications on Cell Survival and Death. *PLoS ONE*. 2009;4: e4407. doi:10.1371/journal.pone.0004407

S12. Zhang X-P, Liu F, Wang W. Two-phase dynamics of p53 in the DNA damage response. *Proc Natl Acad Sci USA*. 2011;108: 8990–8995. doi:10.1073/pnas.1100600108

S13. Zhang X-P, Liu F, Cheng Z, Wang W. Cell fate decision mediated by p53 pulses. *Proc Natl Acad Sci USA*. 2009;106: 12245–12250. doi:10.1073/pnas.0813088106

S14. Zhang X-P, Liu F, Wang W. Coordination between Cell Cycle Progression and Cell Fate Decision by the p53 and E2F1 Pathways in Response to DNA Damage. *J Biol Chem*. 2010;285: 31571–31580. doi:10.1074/jbc.M110.134650

S15. So S, Davis AJ, Chen DJ. Autophosphorylation at serine 1981 stabilizes ATM at DNA damage sites. *J Cell Biol*. 2009;187: 977–990. doi:10.1083/jcb.200906064

S16. Bakkenist CJ, Kastan MB. DNA damage activates ATM through intermolecular autophosphorylation and dimer dissociation. *Nature*. 2003;421: 499–506. doi:10.1038/nature01368

S17. Banin S, Moyal L, Shieh S-Y, Taya Y, Anderson CW, Chessa L, et al. Enhanced Phosphorylation of p53 by ATM in Response to DNA Damage. *Science*. 1998;281: 1674–1677. doi:10.1126/science.281.5383.1674

S18. Canman CE, Lim D-S, Cimprich KA, Taya Y, Tamai K, Sakaguchi K, et al. Activation of the ATM Kinase by Ionizing Radiation and Phosphorylation of p53. *Science*. 1998;281: 1677–1679. doi:10.1126/science.281.5383.1677

S19. Chehab NH, Malikzay A, Stavridi ES, Halazonetis TD. Phosphorylation of Ser-20 mediates stabilization of human p53 in response to DNA damage. *Proc Natl Acad Sci USA*. 1999;96: 13777–13782.

S20. Shieh SY, Taya Y, Prives C. DNA damage-inducible phosphorylation of p53 at N-terminal sites including a novel site, Ser20, requires tetramerization. *EMBO J*. 1999;18: 1815–1823. doi:10.1093/emboj/18.7.1815

S21. Wei C-L, Wu Q, Vega VB, Chiu KP, Ng P, Zhang T, et al. A global map of p53 transcription-factor binding sites in the human genome. *Cell*. 2006;124: 207–219. doi:10.1016/j.cell.2005.10.043

S22. Shieh SY, Ikeda M, Taya Y, Prives C. DNA damage-induced phosphorylation of p53 alleviates inhibition by MDM2. *Cell*. 1997;91: 325–334.

S23. Siliciano JD, Canman CE, Taya Y, Sakaguchi K, Appella E, Kastan MB. DNA damage induces phosphorylation of the amino terminus of p53. *Genes Dev*. 1997;11: 3471–3481.

S24. Maya R, Balass M, Kim S-T, Shkedy D, Leal J-FM, Shifman O, et al. ATM-dependent phosphorylation of Mdm2 on serine 395: role in p53 activation by DNA damage. *Genes Dev*. 2001;15: 1067–1077. doi:10.1101/gad.886901

S25. Winter M, Sombroek D, Dauth I, Moehlenbrink J, Scheuermann K, Crone J, et al. Control of HIPK2 stability by ubiquitin ligase Siah-1 and checkpoint kinases ATM and ATR. *Nat Cell Biol*. 2008;10: 812–824. doi:10.1038/ncb1743

S26. D'Orazi G, Cecchinelli B, Bruno T, Manni I, Higashimoto Y, Saito S, et al. Homeodomain-interacting protein kinase-2 phosphorylates p53 at Ser 46 and mediates apoptosis. *Nat Cell Biol*. 2002;4: 11–19. doi:10.1038/ncb714

S27. Dauth I, Krüger J, Hofmann TG. Homeodomain-interacting protein kinase 2 is the ionizing radiation-activated p53 serine 46 kinase and is regulated by ATM. *Cancer Res*. 2007;67: 2274–2279. doi:10.1158/0008-5472.CAN-06-2884

S28. Hofmann TG, Möller A, Sirma H, Zentgraf H, Taya Y, Dröge W, et al. Regulation of p53 activity by its interaction with homeodomain-interacting protein kinase-2. *Nat Cell Biol*. 2002;4: 1–10. doi:10.1038/ncb715

S29. Tomasini R, Samir AA, Carrier A, Isnardon D, Cecchinelli B, Soddu S, et al. TP53INP1s and Homeodomain-interacting Protein Kinase-2 (HIPK2) Are Partners in Regulating p53 Activity. *J Biol Chem*. 2003;278: 37722–37729. doi:10.1074/jbc.M301979200

S30. Barak Y, Juven T, Haffner R, Oren M. mdm2 expression is induced by wild type p53 activity. *EMBO J*. 1993;12: 461–468.

S31. Kubbutat MHG, Jones SN, Vousden KH. Regulation of p53 stability by Mdm2. *Nature*. 1997;387: 299–303. doi:10.1038/387299a0

S32. Fiscella M, Zhang H, Fan S, Sakaguchi K, Shen S, Mercer WE, et al. Wip1, a novel human protein phosphatase that is induced in response to ionizing radiation in a p53-dependent manner. *Proc Natl Acad Sci USA*. 1997;94: 6048–6053.

S33. el-Deiry WS, Tokino T, Velculescu VE, Levy DB, Parsons R, Trent JM, et al. WAF1, a potential mediator of p53 tumor suppression. *Cell*. 1993;75: 817–825.

S34. Miyashita T, Reed JC. Tumor suppressor p53 is a direct transcriptional activator of the human bax gene. *Cell*. 1995;80: 293–299.

S35. Stambolic V, MacPherson D, Sas D, Lin Y, Snow B, Jang Y, et al. Regulation of PTEN transcription by p53. *Mol Cell*. 2001;8: 317–325.

S36. Shreeram S, Demidov ON, Hee WK, Yamaguchi H, Onishi N, Kek C, et al. Wip1 Phosphatase Modulates ATM-Dependent Signaling Pathways. *Mol Cell*. 2006;23: 757–764. doi:10.1016/j.molcel.2006.07.010

S37. Lu X, Nguyen T-A, Zhang X, Donehower LA. The Wip1 phosphatase and Mdm2: cracking the "Wip" on p53 stability. *Cell Cycle*. 2008;7: 164–168.

S38. Marte BM, Downward J. PKB/Akt: connecting phosphoinositide 3-kinase to cell survival and beyond. *Trends Biochem Sci*. 1997;22: 355–358. doi:10.1016/S0968-0004(97)01097-9

S39. Kandel ES, Hay N. The regulation and activities of the multifunctional serine/threonine kinase Akt/PKB. *Exp Cell Res*. 1999;253: 210–229. doi:10.1006/excr.1999.4690

S40. Franke TF, Yang S-I, Chan TO, Datta K, Kazlauskas A, Morrison DK, et al. The protein kinase encoded by the Akt proto-oncogene is a target of the PDGF-activated phosphatidylinositol 3-kinase. *Cell*. 1995;81: 727–736. doi:10.1016/0092-8674(95)90534-0

S41. Mayo LD, Donner DB. A phosphatidylinositol 3-kinase/Akt pathway promotes translocation of Mdm2 from the cytoplasm to the nucleus. *Proc Natl Acad Sci USA*. 2001;98: 11598–11603. doi:10.1073/pnas.181181198

S42. Haupt Y, Maya R, Kazaz A, Oren M. Mdm2 promotes the rapid degradation of p53. *Nature*. 1997;387: 296–299. doi:10.1038/387296a0

S43. Ogawara Y, Kishishita S, Obata T, Isazawa Y, Suzuki T, Tanaka K, et al. Akt Enhances Mdm2-mediated Ubiquitination and Degradation of p53. *J Biol Chem*. 2002;277: 21843–21850. doi:10.1074/jbc.M109745200

S44. Carracedo A, Pandolfi PP. The PTEN–PI3K pathway: of feedbacks and cross-talks. *Oncogene*. 2008;27: 5527–5541. doi:10.1038/onc.2008.247

S45. Cantley LC. The Phosphoinositide 3-Kinase Pathway. *Science*. 2002;296: 1655–1657. doi:10.1126/science.296.5573.1655
