# Supplementary figures and images for: Feedbacks, Bifurcations, and Cell Fate Decision-Making in the p53 System
Source: PLoS Comput Biol. 2016 Feb 29;12(2):e1004787. doi: 10.1371/journal.pcbi.1004787 (PMC4771203; doi:10.1371/journal.pcbi.1004787)

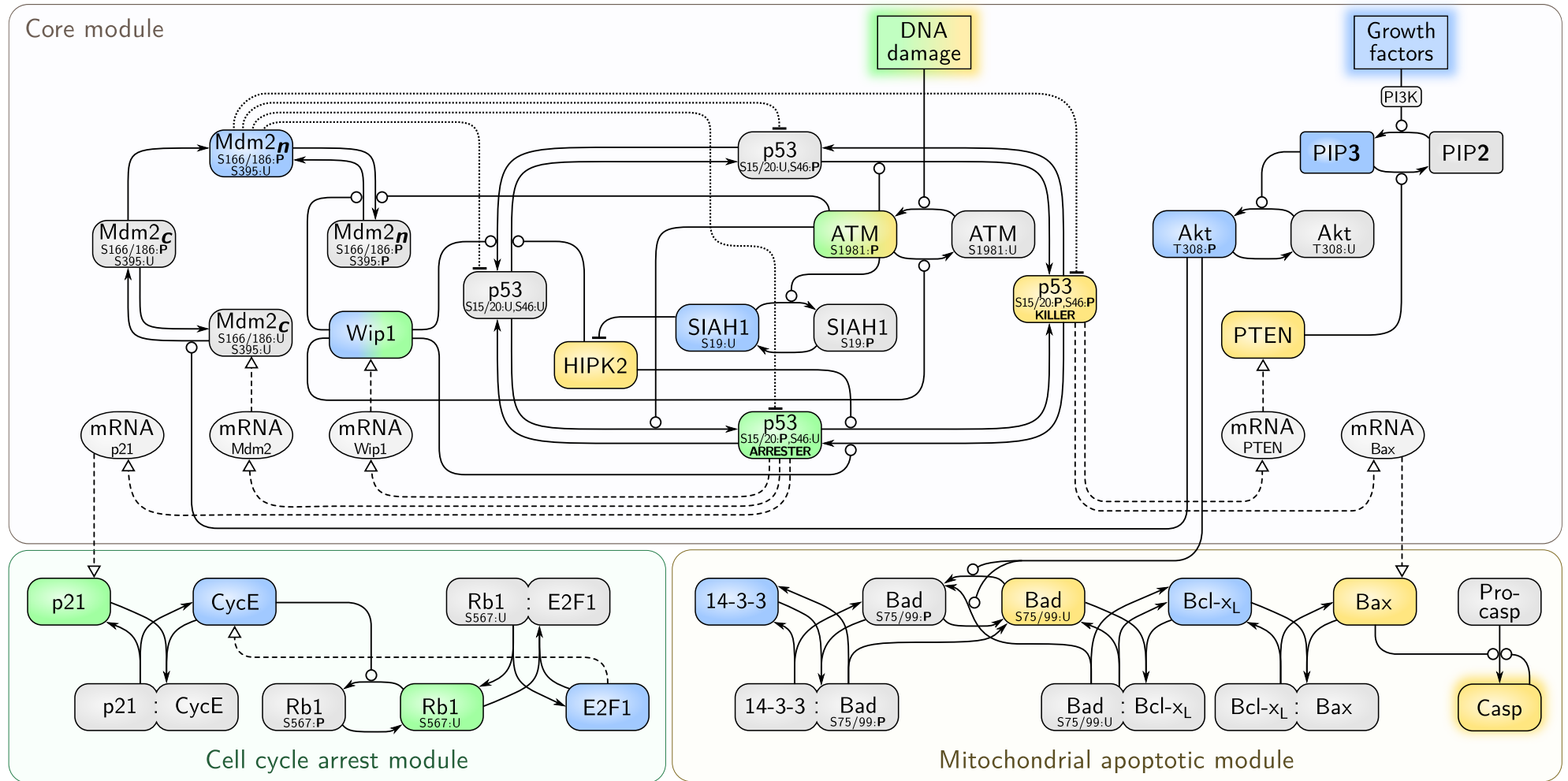

Supplement: S1 Fig — Arrow-headed dashed lines indicate transcriptional regulation, arrow-headed solid lines—protein transformation, circle-headed solid lines—positive influence, hammer-headed dotted lines—ubiquitination by Mdm2 leading to protein degradation. The subscripts n or c denote either nuclear or cytoplasmic localization of Mdm2. Bold ‘P’ and non-bold ‘U’ denote phosphorylated and unphosphorylated states of given residues, respectively. Pro-survival and cycle-promoting proteins are represented with blue boxes, pro-apoptotic proteins with yellow boxes, proteins involved in cell cycle arrest with green boxes, while the remaining proteins and protein complexes are left in grey boxes. (PDF) [file pcbi.1004787.s002.pdf]
