# Supplementary material for: Feedbacks, Bifurcations, and Cell Fate Decision-Making in the p53 System
Source: PLoS Comput Biol. 2016 Feb 29;12(2):e1004787. doi: 10.1371/journal.pcbi.1004787 (PMC4771203; doi:10.1371/journal.pcbi.1004787)
Supplement: S2 Fig — Trajectories for p53 and Mdm2 were simulated using the model variant in which there is no Wip1-mediated dephosphorylation of ATM. Simulations were performed for four different pairs of Wip1 (s1) and PTEN (s2) synthesis rates, with IR dose equal 3 Gy. Black line corresponds to nominal values of s1 and s2. (PDF) [file pcbi.1004787.s003.pdf]

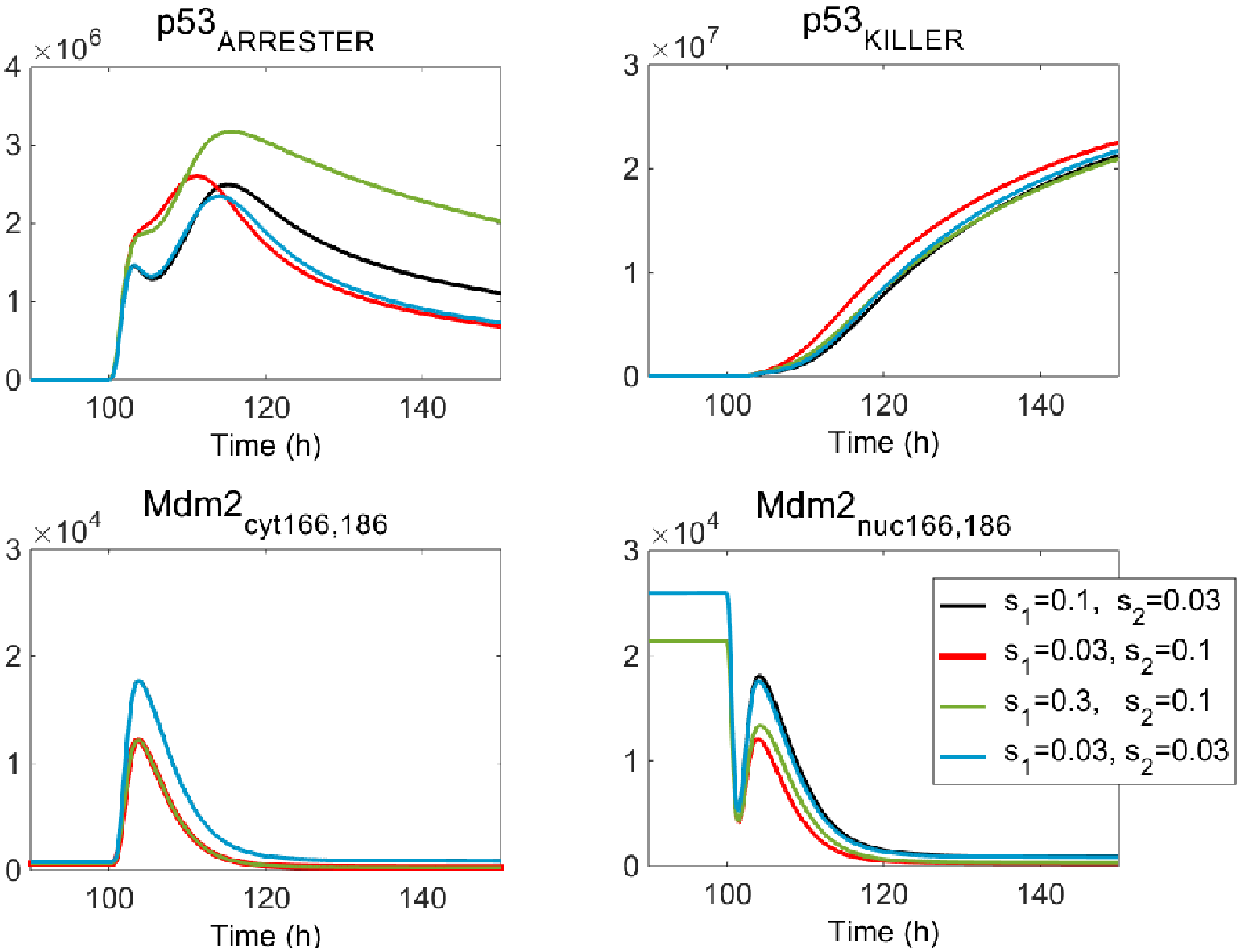

S2 Figure: The negative feedback mediated by Wip1 is required for generating oscillations. Trajectories for p53 and Mdm2 were simulated using the model variant in which there is no Wip1-mediated dephosphorylation of ATM. Simulations were performed for four different pairs of Wip1 (s_1) and PTEN (s_2) synthesis rates, with IR dose equal 3 Gy. Black line corresponds to nominal values of s_1 and s_2.
