# Supplementary material for: Feedbacks, Bifurcations, and Cell Fate Decision-Making in the p53 System
Source: PLoS Comput Biol. 2016 Feb 29;12(2):e1004787. doi: 10.1371/journal.pcbi.1004787 (PMC4771203; doi:10.1371/journal.pcbi.1004787)
Supplement: S3 Fig — PTEN mRNA synthesis rate is equal to the nominal value s2 = 0.03; Wip1 synthesis rate is equal s1 = 0.2 in (B) and s1 = 0.1 in (C). The number of DSBs is equal 100 for (A) and (B). The stable and unstable steady states are indicated by solid and dashed lines, respectively. Dots and open circles show the maxima and minima of the stable and unstable limit cycles, respectively. Green vertical line shows the Neimark—Sacker bifurcation (N—S). Red dots mark saddle—node bifurcations (SN1, SN2), yellow dots mark the supercritical Hopf (Hsuper) and the subcritical Hopf (Hsub) bifurcation. Note the log-scale on the vertical axis. The bifurcation diagrams with respect to Wip1 and PI3K resemble the mirror image of the bifurcation diagram with respect to PTEN (see main text Fig 5A). The bifurcation diagram with respect to DNA damage is similar to the bifurcation diagram with respect to PTEN, but the limit cycle oscillations start at non-zero value of the bifurcation parameter. (PDF) [file pcbi.1004787.s004.pdf]

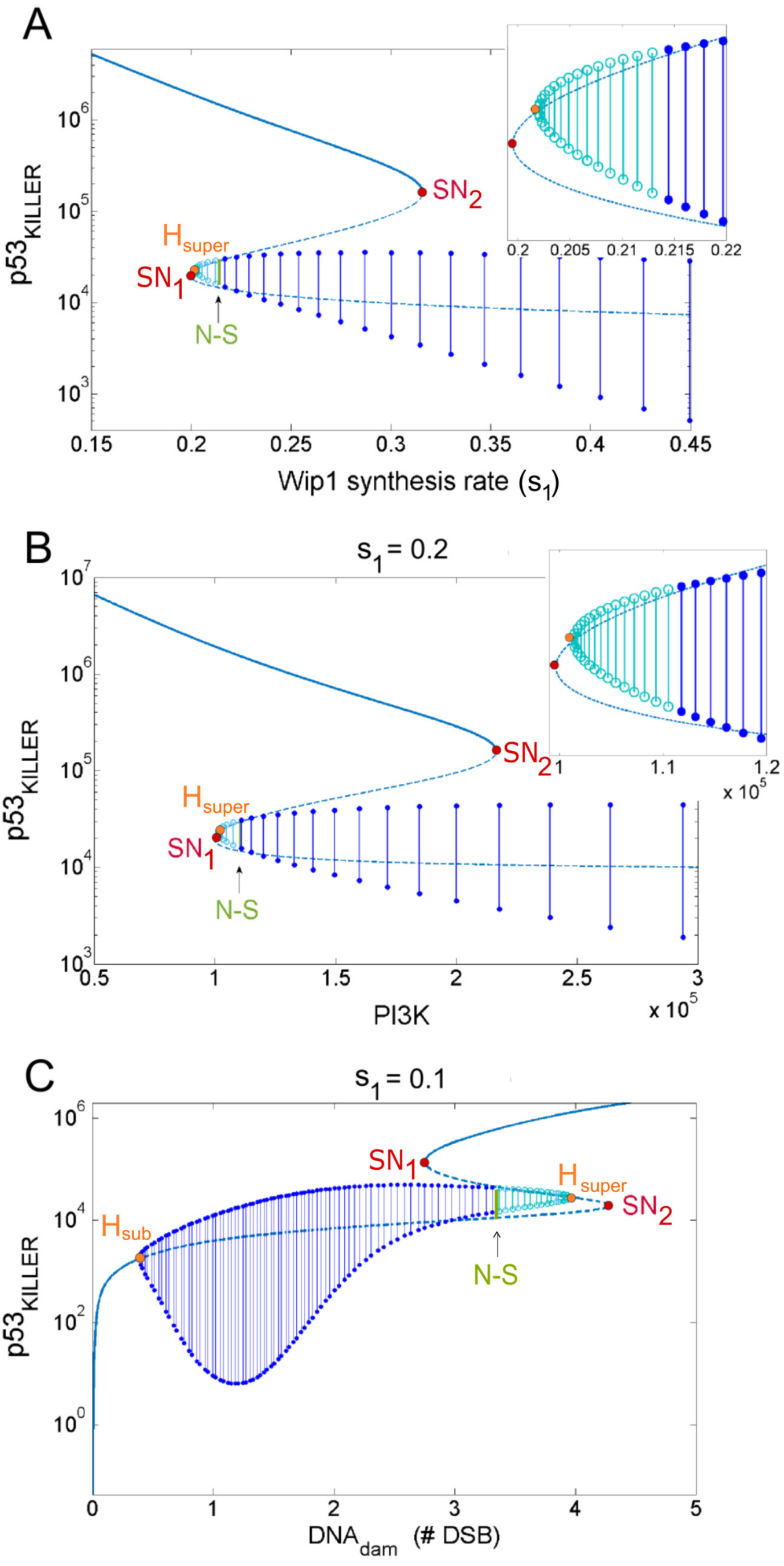

S3 Figure: Recurrent solutions for p53$_{\mathrm{KILLER}}$ as a function of Wip1 synthesis rate, active PI3K level and DNA damage level. PTEN mRNA synthesis rate is equal to the nominal value $s_2 = 0.03$; Wip1 synthesis rate is equal $s_1 = 0.2$ in (**B**) and $s_1 = 0.1$ in (**C**). The number of DSBs is equal 100 for (**A**) and (**B**). The stable and unstable steady states are indicated by solid and dashed lines, respectively. Dots and open circles show the maxima and minima of the stable and unstable limit cycles, respectively. Green vertical line shows the Neimark–Sacker bifurcation (N–S). Red dots mark saddle–node bifurcations (SN_1, SN_2), yellow dots mark the supercritical Hopf ($\mathrm{H}_{\mathrm{super}}$) and the subcritical Hopf ($\mathrm{H}_{\mathrm{sub}}$) bifurcation. Note the log-scale on the vertical axis. The bifurcation diagrams with respect to Wip1 and PI3K resemble the mirror image of the bifurcation diagram with respect to PTEN (see main text Fig 5A). The bifurcation diagram with respect to DNA damage is similar to the bifurcation diagram with respect to PTEN, but the limit cycle oscillations start at non-zero value of the bifurcation parameter.
