# Supplementary material for: Feedbacks, Bifurcations, and Cell Fate Decision-Making in the p53 System
Source: PLoS Comput Biol. 2016 Feb 29;12(2):e1004787. doi: 10.1371/journal.pcbi.1004787 (PMC4771203; doi:10.1371/journal.pcbi.1004787)
Supplement: S4 Fig — (A) and (B): nominal gene switching rates. (C) and (D): 10-fold increased gene switching rates. The irradiation phase started at Time = 300 h and lasted for 10 min, the irradiation dose was 4 Gy. The dynamics of the p53tot and Mdm2tot weakly depends on the gene switching rate, although its increase leads to some decrease of the amplitude of fluctuations in unstimulated cells. Notice the logarithmic scale on vertical axes. (PDF) [file pcbi.1004787.s005.pdf]

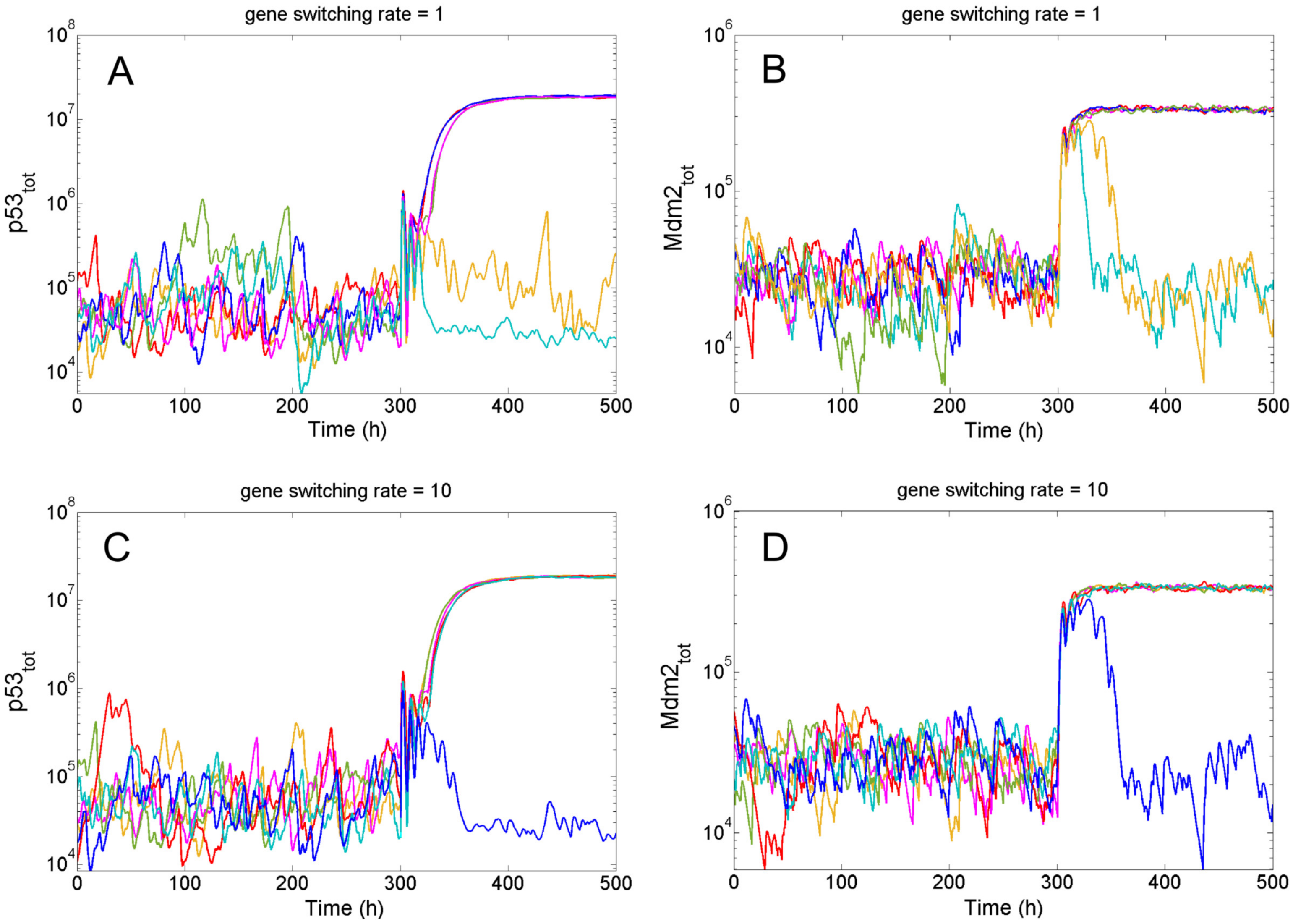

S4 Figure: Influence of the gene switching rates on single-cell stochastic trajectories for $p53_{tot}$ and $Mdm2_{tot}$. (**A**) and (**B**): nominal gene switching rates. (**C**) and (**D**): 10-fold increased gene switching rates. The irradiation phase started at Time = 300 h and lasted for 10 min, the irradiation dose was 4 Gy. The dynamics of the $p53_{tot}$ and $Mdm2_{tot}$ weakly depends on the gene switching rate, although its increase leads to some decrease of the amplitude of fluctuations in unstimulated cells. Notice the logarithmic scale on vertical axes.
