# Supplementary material for: Feedbacks, Bifurcations, and Cell Fate Decision-Making in the p53 System
Source: PLoS Comput Biol. 2016 Feb 29;12(2):e1004787. doi: 10.1371/journal.pcbi.1004787 (PMC4771203; doi:10.1371/journal.pcbi.1004787)
Supplement: S5 Fig — The stable limit cycle (of envelope marked by pairs of blue points) coexists with a high-level stable steady state and disappears through the saddle—loop bifurcation (SL). The stable and unstable steady states are indicated by solid and dashed lines, respectively; red dots labeled SN mark saddle—node bifurcations. (PDF) [file pcbi.1004787.s006.pdf]

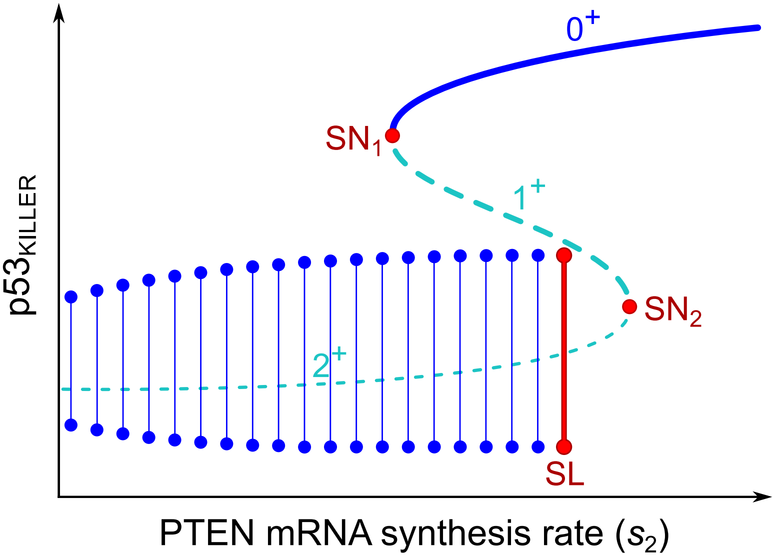

S5 Figure: A hypothetical alternative bifurcation structure for the core module. The stable limit cycle (of envelope marked by pairs of blue points) coexists with a high-level stable steady state and disappears through the saddle–loop bifurcation (SL). The stable and unstable steady states are indicated by solid and dashed lines, respectively; red dots labeled SN mark saddle–node bifurcations.
